# Comparative Bioinformatics Analysis of Transcription Factor Genes Indicates Conservation of Key Regulatory Domains among *Babesia bovis*, *Babesia microti*, and *Theileria equi*

**DOI:** 10.1371/journal.pntd.0004983

**Published:** 2016-11-10

**Authors:** Heba F. Alzan, Donald P. Knowles, Carlos E. Suarez

**Affiliations:** 1 Parasitology and Animal Diseases Department, National Research Center, Dokki, Giza, Egypt; 2 Animal Disease Research Unit, Agricultural Research Service, USDA, WSU, Pullman, Washington, United States of America; 3 Department of Veterinary Microbiology and Pathology, Washington State University, Pullman, Washington, United States of America; University of California, School of Medicine, UNITED STATES

## Abstract

Apicomplexa tick-borne hemoparasites, including *Babesia bovis*, *Babesia microti*, and *Theileria equi* are responsible for bovine and human babesiosis and equine theileriosis, respectively. These parasites of vast medical, epidemiological, and economic impact have complex life cycles in their vertebrate and tick hosts. Large gaps in knowledge concerning the mechanisms used by these parasites for gene regulation remain. Regulatory genes coding for DNA binding proteins such as members of the *Api-AP2*, *HMG*, and *Myb* families are known to play crucial roles as transcription factors. Although the repertoire of Api-AP2 has been defined and a *HMG* gene was previously identified in the *B*. *bovis* genome, these regulatory genes have not been described in detail in *B*. *microti* and *T*. *equi*. In this study, comparative bioinformatics was used to: (i) identify and map genes encoding for these transcription factors among three parasites’ genomes; (ii) identify a previously unreported *HMG* gene in *B*. *microti*; (iii) define a repertoire of eight conserved *Myb* genes; and (iv) identify AP2 correlates among *B*. *bovis* and the better-studied *Plasmodium* parasites. Searching the available transcriptome of *B*. *bovis* defined patterns of transcription of these three gene families in *B*. *bovis* erythrocyte stage parasites. Sequence comparisons show conservation of functional domains and general architecture in the AP2, Myb, and HMG proteins, which may be significant for the regulation of common critical parasite life cycle transitions in *B*. *bovis*, *B*. *microti*, and *T*. *equi*. A detailed understanding of the role of gene families encoding DNA binding proteins will provide new tools for unraveling regulatory mechanisms involved in *B*. *bovis*, *B*. *microti*, and *T*. *equi* life cycles and environmental adaptive responses and potentially contributes to the development of novel convergent strategies for improved control of babesiosis and equine piroplasmosis.

## Author Summary

The tick-borne apicomplexan parasites *Babesia* and *Theileria* are responsible for costly and devastating diseases globally. Improved control is needed, but the biology of these parasites remains poorly understood. Significant gaps include better understanding of the mechanisms involved in control of gene expression and the events leading to parasite development among hosts, including the production of sexual stages in their definitive tick vector hosts. Similar to other better-studied eukaryotic cells, it is likely that regulatory genes coding for DNA binding proteins such as members of the *Api-AP2*, *HMG*, and *Myb* families play crucial roles as transcription factors in these processes, but these genes remain uncharacterized in these three related parasites. In this study, we describe the presence and genomic organization of these three types of genes in *Babesia bovis*, *Babesia microti*, and *Theileria equi*, highlighting the importance of the conservation of these genes and their possible contributions to parasite development through their different life stages. We also describe the occurrence of a previously unreported *HMG* gene in *B*. *microti*, an important emerging human pathogen; define the repertoire of eight conserved *Myb* genes; and describe the pattern of transcription of the regulatory *AP2*, *HMG*, and *Myb* genes in *B*. *bovis* intra-erythrocytic stages for the first time. It is expected that these findings will elicit additional research in this field and contribute to the development of converged intervention strategies for the improved control of these devastating and generally under-studied diseases.

## Introduction

The tick-borne apicomplexan intraerythrocytic parasites *Babesia bovis*, *Babesia microti*, and *Theileria equi* cause similar potentially fatal acute hemolytic disease and persistent infections in bovines, humans, and equids, respectively. *B*. *bovis* and *T*. *equi* are mainly transmitted by *Rhiphicephalus* ticks, whereas *B*. *microti* is primarily transmitted by *Ixodes scapularis* [1]. Despite the use of tick control measures, the availability of live vaccines for preventing acute disease caused by *B*. *bovis*, as well as effective chemotherapeutics, bovine babesiosis and equine theileriosis remain poorly controlled globally. Both *B*. *bovis* and *T*. *equi* are responsible for large economic losses, while *B*. *microti* is responsible for public health concerns. These related apicomplexan parasites are able to cause persistent infections and have achieved a high degree of adaptation through millions of years of co-evolution within their tick and mammal hosts, resulting in the development of complex survival strategies. A practical consequence of these natural evolutionary processes is that the development of control measures against these parasites is extremely difficult to achieve [2]. Clearly, an improved understanding of the biology of *Babesia* and *Theileria* parasites is needed for designing novel and improved methods of control. However, important gaps of knowledge remain in our understanding of the biology of these parasites and the molecular mechanisms involved in interactions with their mammal and tick hosts [2]. Mining of genomes of *B*. *bovis* [3], *B*. *microti* [4] [5], and *T*. *equi* [6], based on known regulatory mechanisms used by eukaryotic cells, combined with current high-throughput research technologies such as transcriptomics, proteomics, metabolomics, gene editing, and transfection systems, can be employed to understand complex gene expression regulatory networks. Regulation of gene expression in eukaryotic cells can be achieved at the transcriptional level using both genetic and epigenetic mechanisms. Moreover, it is likely that the activity of transcription factors and DNA binding proteins, combined and acting in coordination with modulated chromatin organizations such as nucleosome positioning, essentially controls gene expression at different parasite life cycle stages [7]. In addition, gene expression can also be regulated at the post-transcriptional and translational levels. Key advances in understanding mechanisms involved in gene regulation have so far been achieved in the more studied *Babesia*-related *Plasmodium*, *Theileria annulata*, and *Toxoplasma*, among others [8]. These studies serve as a model for characterization of similar and generally conserved gene regulatory mechanisms in *Babesia* and closely related parasites.

Intriguingly, genomic and proteomic analysis initially performed in *Plasmodium* showed a paucity of genes encoding for recognizable and typical enhancers and transcription activators, such as transcription factors (TFs), despite the need for coordinated regulation of gene expression for parasite survival in dramatically different life stages [9–11]. These observations support the hypothesis of the evolution of unique transcription factors in *Plasmodium* parasites. These insights prompted recent investigations in *Plasmodium* and other related apicomplexans, leading to the identification and characterization of at least three well-characterized TFs: proteins encoded by the apicomplexan AP2 gene family (ApiAP2) and the Myb and HMG proteins [10]. This study describes the conservation of genes encoding for these three types of gene transcriptional regulators in *B*. *bovis*, *B*. *microti*, and *T*. *equi* parasites. The best characterized of these three factors is the AP2 gene family. This family is related to the Apetala 2 gene family originally identified in plants encoding for proteins that are involved in the regulation of transcription in many crucial cell stage developmental transitions. The plant AP2/ERF (Apetela2/Ethylene Response Factor) gene family [12] encodes a diverse family of proteins containing one or two ~60 amino acid conserved AP2 domains. Consistent with their function as nuclear transcriptional factors, the AP2 domains contain conserved structural motifs that are directly involved in DNA binding. Thus, the AP2 domains can bind DNA in a sequence-specific fashion. Initial studies suggested the involvement of an 18 amino acid core region that forms an amphipathic α-helix [13], but further structural studies demonstrated that Ap2 proteins use an antiparallel three-stranded β-sheet to make major groove contacts. The majority of the DNA contacts are made by arginine and tryptophan residues located in the β-sheet of the AP2 domains of the protein [14]. Further structural analysis in the *Plasmodium falciparum* PF14_0633 AP2 domain also revealed that a β-sheet fold binds the DNA major groove through base-specific and backbone contact. The role of the conserved α-helix is stabilization of the triple-stranded β-sheet [9, 15].

The AP2 proteins of plants contain one or two tandem-arranged AP2 domains, which are separated typically by a 25 amino acid conserved linker sequence. However, high sequence divergence was typically found among the regions of AP2 proteins that do not contain the otherwise conserved AP2 domains [16, 17]. Genes encoding for proteins containing AP2 domains were also annotated in the *B*. *bovis*, *T*. *equi*, and *B*. *microti* genomes, but a systematic and detailed characterization of these gene families was not reported for these parasites. In this study, bioinformatics tools are used to define the AP2 repertoire in *T*. *equi* and *B*. *microti* and to find possible AP2 correlates among *B*. *bovis* and *Plasmodium* parasites.

The *Myb* genes are evolutionarily conserved amongst eukaryotic cells. The Myb proteins, which are part of the tryptophan cluster family, are able to bind to DNA and typically regulate genes involved in cell proliferation and differentiation [17]. Myb proteins usually contain two or three distinct DNA binding domain located in their amino terminal regions. The Myb proteins generally contain three repeats of approximately 50 residues with three regularly spaced tryptophan residues [18].

The high mobility group (HMG) box (HMGB) are abundant, small (under 100 amino acids long), non-histone architectural chromosomal proteins. The HMGBs are also highly conserved across eukaryotic cells and have been implicated in a number of basic cellular functions such as DNA replication, transcription, and recombination, perhaps by modulating chromatin structure [19]. Importantly, *Plasmodium* HMGBs have been reported to be differentially expressed and co-related to the development of erythrocyte stages and gametocyte differentiation [20] [21], suggesting their role in parasite differentiation. Interestingly, secretory forms of *Plasmodium* HMG may be involved in triggering host inflammatory immune responses associated with malaria, since they can stimulate macrophages to release cytokines, such as TNF alpha [19], and so they were also implicated in pathogenic mechanisms.

Here, the organization and features of the AP2 gene family in *B*. *bovis* are described. In addition, we compare the features of the *B*. *bovis* AP2 genes with the previously undefined AP2 genes identified in the genome of the related *T*. *equi* and *B*. *microti* tick-borne intra-erythrocytic apicomplexans for the first time, and describe the pattern of expression of the AP2 genes within intra-erythrocyte stage parasites based on previously reported transcriptomic studies in a *B*. *bovis* T2bo strain virulent and attenuated pair [19]. Also discussed is the occurrence and characteristics of genes encoding for Myb and HMG DNA-binding proteins, the two additional classes of transcription factors that were previously identified and assigned significant roles in related parasites, but remained uncharacterized in *B*. *bovis*, *T*. *equi*, and *B*. *microti*.

### The AP2 gene family in *B*. *bovis*, *B*. *microti*, *and T*. *equi*

**Fig 1:**
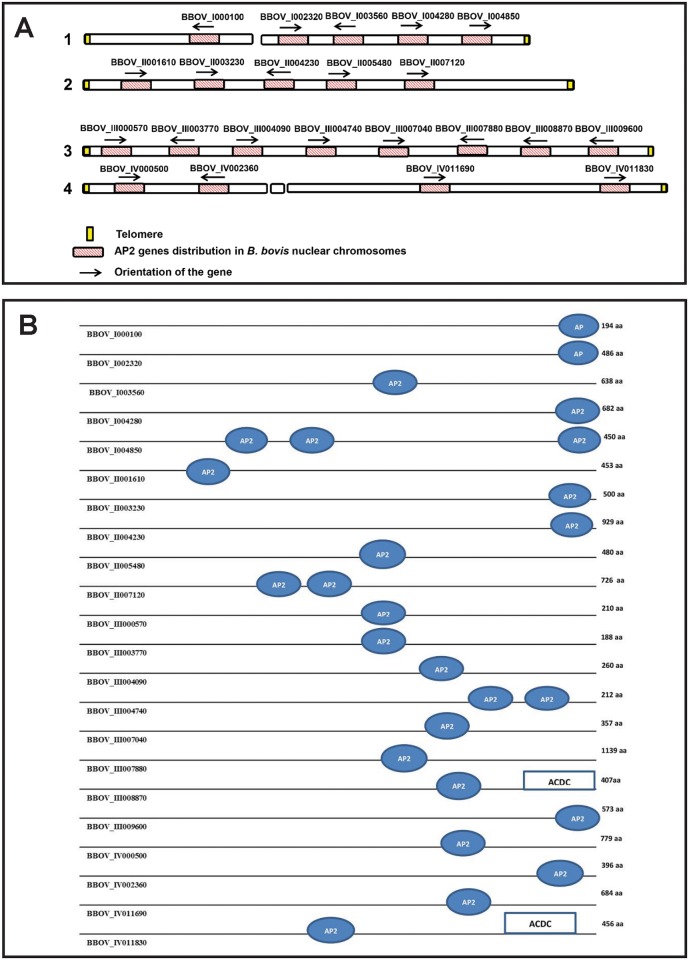
Schematic representation of the location of the 22 AP2 genes and the number and domains identified in the genome of *B*. *bovis* (data not presented to scale). (A) The figure represents the genome localization of the *B*. *bovis* AP2 genes, distributed among the four chromosomes. Each gene orientation is indicated by black arrows. (B) Gene nomenclature, schematic representation of the number and location of the AP2 domains of the *B*. *bovis* AP2 proteins. The presence and location of the ACDC domains in the protein are also indicated.

The presence of AP2 genes in apicomplexans was initially described by Balaji et al. [22], who first reported the identification of members of the AP2 gene family in the genomes of *Plasmodium*, *Theileria*, *Cryptosporidium*, and *Toxoplasma*. Initial genome characterization in the *B*. *bovis* T2Bo strain genome resulted in the annotation of 18 genes encoding for AP2 domain-containing proteins [3]. However, Oberstaller et al. [8], using a highly sensitive Hidden Marcov Model (HMM), recently identified four additional genes encoding for AP2 proteins, thus extending the number of genes encoding for AP2 domain-containing proteins to a total of 22. General features of the 22 *B*. *bovis* genes and their predicted proteins are shown in Table 1. Because AP2 proteins may have more than a single AP2 domain, the *B*. *bovis* AP2 proteins display a total of 26 known AP2 domains. Similar to what was found in other apicomplexan genomes, the AP2 genes are not organized in clusters but dispersed throughout the four chromosomes of *B*. *bovis* (Fig 1A and 1B and Table 1).

**Table 1:** Characteristics of AP2 genes identified in the *B*. *bovis* genome.

Chromosome No.	Gene (Locus tag)	Annotation	Length(gDNA/cDNA/aa)	No. of Exons	No. of AP2 domains	Other conserved domains	PI-MW (kDa)
**Chromosome 1**	BBOV_I000100	hypothetical protein	775bp/585bp/194aa	4	One (110–162)	_	10.23–22.85
	BBOV_I002320	hypothetical protein	1461bp/486aa	1	One (397–441)	_	5.62–65.12
	BBOV_I003560	hypothetical protein	2076bp/638aa	1	One (421–460)	_	5.15–70.06
	BBOV_I004280	hypothetical protein	2049bp/682aa	1	One (609–655)	_	6.08–77.30
	BBOV_I004850	hypothetical protein	1366bp/450aa	1	Three (94–143/166–212/372–420)	_	6.62–52.12
**Chromosome 2**	BBOV_II001610	hypothetical protein	1362bp/453aa	1	One (68–120)	_	5.72–50.22
	BBOV_II003230	hypothetical protein	1532bp/500aa	1	One (408–458)	-PBP1, -PAT1 Amelogenin	7.02–57.40
	BBOV_II004230	hypothetical protein	3339bp/929aa	1	One (823–873)	_	6.15–103.56
	BBOV_II005480	hypothetical protein	1763bp/480aa	1	One (249–298)	_	5.68–54.23
	BBOV_II007120	hypothetical protein	2181bp/726aa	1	Two (187–240/261–317)	-	5.36–82.22
**Chromosome 3**	BBOV_III000570	hypothetical protein	843bp/633bp/210aa	5	One (107–162)	_	9.53–24.92
	BBOV_III003770	hypothetical protein	567bp/188aa	1	One (65–120)	_	9.37–21.87
	BBOV_III004090	hypothetical protein	882bp/260aa	1	One (187–235)	_	11.21–30.50
	BBOV_III004740	hypothetical protein	707bp/639bp/212aa	3	Two (100–145/162–210)	_	9.67–24.27
	BBOV_III007040	hypothetical protein	1074bp/357aa	1	One (202–252)	PRK12937	8.44–40.19
	BBOV_III007880	hypothetical protein	3423bp/1139aa	1	One (537–590)		6.43–130.70
	BBOV_III008870	hypothetical protein	2122bp/1712bp/407aa	5	One (211–264)	ACDC	5.21–45.47
	BBOV_III009600	hypothetical protein	2093bp/1722bp/573aa	1	One (515–564)	_	5.82–63.38
**Chromosome 4**	BBOV_IV000500	hypothetical protein	2340bp/779aa	1	One (493–544)	_	5.71–86.15
	BBOV_IV002360	hypothetical protein	1650bp/396aa	4	One (270–321)	_	5.81–46.26
	BBOV_IV011690	hypothetical protein	2055bp/684aa	2	One (528–583)	_	5.70–75.35
	BBOV_IV011830	hypothetical protein	1371bp/456aa	1	One (170–221)	ACDC	5.2–51.28

Bioinformatics analysis performed on the predicted amino acid sequences of the *B*. *bovis* AP2 proteins shows that some contain other additional known functional domains (Table 1, Fig 1B), such as the ACDC domain (AP2 coincident domain present mostly at the C-terminus of the proteins), a conserved PBP1domain (PAB1-binding protein 1), which is also present in proteins interacting with a poly(A)-binding protein, and in the Topoisomerase II-associated protein (PAT1), a protein that facilitates accurate chromosome separation during cell division (Table 1). Consistently, and together with the AP2 domain, all these additional domains are known to function in a nuclear environment. Predicted intracellular localization and routing of *B*. *bovis* AP2 proteins into the cell nucleus is consistent with the lack of signal peptides in all the putative *B*. *bovis* AP2 proteins as determined by sequence analysis using the SMART programs (http://smart.embl-heidelberg.de/smart/set_mode.cgi?NORMAL=1). In addition, cellular localization predictions using the program Cello v2.5 (http://cello.life.nctu.edu.tw/) predicted an intranuclear subcellular localization for all *B*. *bovis* AP2 proteins.

The predicted molecular size and isoelectric points of the *B*. *bovis* AP2s are also highly diverse, ranging from ~21 to 103 kDa to 5.15 to 11.21 kDa (Table 1). In general, there appears to be an association between isoelectric points (pI) and size of the molecules, and, thus, molecules with higher pI are of a relatively smaller size than the ones with a lower pI (Table 1). This association is consistent with a previous study by Kiraga et al. [23], although its biological relevance remains unknown.

While 19 out of the 22 known *B*. *bovis* AP2 proteins contain a single AP2 domain, the genes BBOV_II007120 and BBOV_III004740 contain two AP2 domains, and gene BBOV_I004850 has three AP2 domains (Table 1, Fig 1B). Similar to AP2 proteins in plants, two of the three domains in the putative protein encoded by BBOV_I004850 are separated by 25 amino acids in the amino terminal part of the molecule, whereas the third domain is distally localized, separated by 160 amino acids from the second domain and 30 amino acids apart from the C-terminal end of the molecule. The AP2 protein encoded by gene BBOV_II007120 contains the two AP2 domains separated by 21 amino acids, whereas the two AP2 domains of the protein encoded by gene BBOV_III004740 are just 17 amino acids apart. It is possible that proteins containing multiple AP2 domains are able to bind to distinct DNA regions either separately or simultaneously, thus adding increasing functional versatility for these molecules. In general, and consistent with what was found for other AP2 proteins, there is low sequence identity or similarity among the AP2 proteins, and, thus, their similarities are just restricted to the conserved 60 amino acid domain [8, 22, 24]. The percent identities found among the full AP2 proteins after their alignment is shown in S1 Table. The alignment and the identity results were obtained by using Clustal omega (http://www.ebi.ac.uk/Tools/msa/clustalo/). The more significantly related AP2 proteins are BBOV_I004850 and BBOV_II005480, sharing 25.59% identity (S1 Table), followed by BBOV_I000100 and BBOV_III003770, with 23.68% identity (S1 Table). Overall, these data suggest that, with few exceptions, the *B*. *bovis* AP2 proteins are not highly related in sequence outside the AP2 domains.

Alignments of the AP2 domain among all *B*. *bovis* revealed 100% identity between the AP2 domains in BBOV_III003770 and BBOV_I003560, suggesting the possibility of shared DNA binding specificities. Interestingly, the highly related domains BBOV_I004850.3 and BBOV_I004850.2 (sharing 51.02% identity) are both localized in the same protein (gene BBOV_I004850).

**Fig 2:**
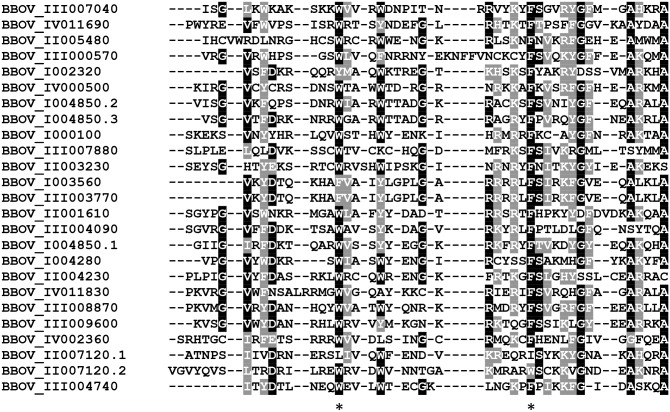
Sequence alignment of all AP2 domains found in the 22 *B*. *bovis* AP2 proteins. Gene denominations are indicated on the left. Numerations (1–3) indicate the number of domains in each protein. Similar and identical amino acid residues are indicated in gray and black font, respectively. The [*] indicates conservation of W and F amino acids residues among the *B*. *bovis* AP2 domains.

Alignments among all *B*. *bovis* AP2 domains (Fig 2) show that certain amino acid residues have a high degree of sequence conservation and may be functionally required in the *B*. *bovis* AP2 proteins. For instance, and similar to what was found for other apicomplexan AP2 proteins, all *B*. *bovis* AP2 domains contain highly conserved W and F residues (labeled with asterisks in Fig 2). It is known that these positional conserved residues are likely to help stabilize hydrophobic interactions between the AP2 domain and its recognized DNA target [22]. Consistently, and as described in more detail below, these residues are also conserved in the AP2 domains identified in the *B*. *bovis* related intra-erythrocytic apicomplexan *B*. *microti* and *T*. *equi* (S1 and S2 Figs). In addition, other amino acids are also highly conserved (Fig 2) among the *B*. *bovis* AP2 domains.

**Fig 3:**
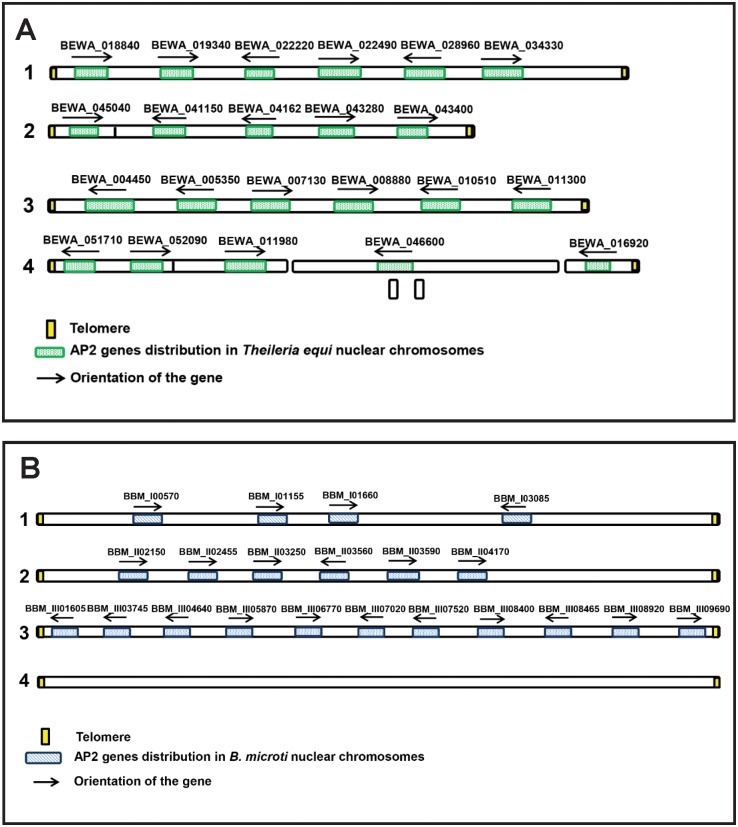
(A) Schematic representation of the location of the 22 AP2 genes identified in the genome of *T*. *equi* (data not presented in scale). (B) Schematic representation of the location of the 21 AP2 genes identified in the genome of *B*. *microti* (data not presented in scale).

Just 20 AP2 genes were annotated as containing AP2 domains in the published *T*. *equi* genome [6]. However, using further bioinformatics analysis, we found that genes BEWA_041620 and BEWA_018840 also contain AP2 domains. Thus, we propose that *T*. *equi* contains at least 22 AP2 genes. The organization and orientation of such genes into the four nuclear *T*. *equi* chromosomes are depicted in Fig 3A and S2 Table. Similar to what was observed for *B*. *bovis*, the *T*. *equi* AP2 genes are scattered among all four chromosomes (Fig 3A). As it was found for *B*. *bovis*, the AP2 genes of *T*. *equi* may also contain 1, 2 or 3 AP2 domains.

Similar searches performed on the published *B*. *microti* genome [5] resulted in the identification of 21 AP2 genes (S3 Table). All the Ap2 domain-containing genes present in the *B*. *microti* genome were previously annotated as such, except gene BBM_III08920 coding for a protein with a single previously unnoticed Ap2 domain, which is reported here for the first time. Fig 3B describes the organization as well as the orientation of the 21 AP2 genes into the four chromosomes of *B*. *microti*. Similar to what was found for *B*. *bovis*, the *T*. *equi* and *B*. *microti* AP-2 proteins contain other conserved domains, such as the ACDC and the PBP1 domains (S2 and S3 Tables).

Sequence comparisons among all the Ap2 domains identified in the *B*. *bovis*, *T*. *equi*, and *B*. *microti* putative AP2 proteins (Table 2) revealed high levels of identity among some domains. The identity reaches 100% among domains from proteins BBOV_III008870 (*B*. *bovis*), and BEWA_010510 (*T*. *equi*), and BBM_III06770 (*B*. *microti*). Interestingly, the proteins encoded by the *B*. *bovis* gene BBOV_I004850 and the *T*. *equi* BEWA_011980 gene have three highly similar domains. They share 100% identity for their first domain, which is also highly conserved in the *B*. *microti* protein encoded by gene BBM_III05870.1 (95.74% identity). Additional domain similarities are described in Table 2.

**Table 2:** Identity percent of selected Ap2 domains among *B*. *bovis*, *B*. *microti*, and *T*. *equi*.

		*B*. *bovis* domain						*T*. *equi* domain					
		BBOV_III008870	BBOV_I004850.1	BBOV_I004850.2	BBOV_I004850.3	BBOV_II005480	BBOV_III007040	BEWA_010510	BEWA_011980.1	BEWA_011980.2	BEWA_011980.3	BEWA_022490	BEWA_007130
***T*. *equi* domain**	BEWA_010510	100											
	BEWA_011980.1		100										
	BEWA_011980.2			93.88									
	BEWA_011980.3				83.33								
	BEWA_022490					93.88							
	BEWA_007130						94.12						
***B*. *microti* domain**	BBM_III06770	100						100					
	BBM_III05870.1		95.74						95.74				
	BBM_III05870.2			89.8						89.8			
	BBM_I03085				NH	84					NH	84	NH

NH, No significant homology

The functions and DNA-binding specificities of the *B*. *bovis*, *B*. *microti*, and *T*. *equi* AP2 domains remain unknown, and they will need to be defined experimentally. Remarkably, the specificity of binding of some AP2 proteins to certain short DNA target motifs (usually six to seven base-pairs long) appears to be quite conserved among distinct *Plasmodium* species and, furthermore, among other related apicomplexans [7, 25]. These findings suggest that *Plasmodium* binding specificity data together with bioinformatics analysis on the 5′ upstream gene coding regions could guide the design of future experiments aimed at establishing the DNA binding specificities of the AP2 proteins in the three parasites examined in this study.

**Fig 4:**
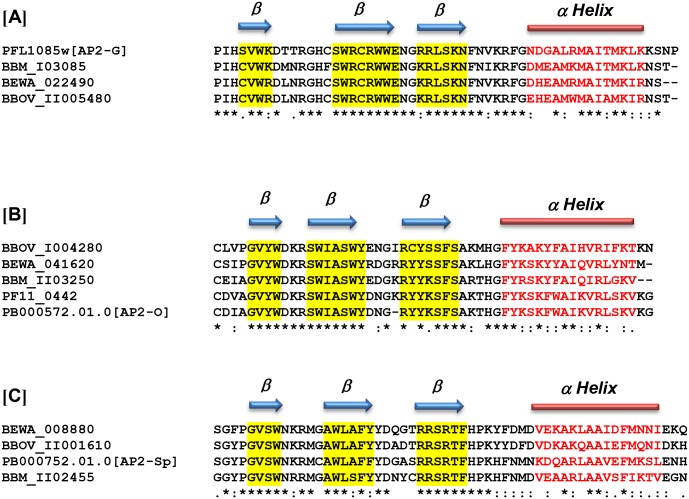
Alignments among *B.* *bovis*, *B*. *microti*, *T equi* and functionally defined *Plasmodium* Ap2 domains. Alignments of: (**A)** *B*. *bovis* BBOV_II005480, *B*. *microti* BBM_I03085, and *T*. *equi* BEWA_022490 with *P*. *berghei* PB000752.01.0, a domain involved in the development of sexual-stage forms in *Plasmodium* (AP2-G). **(B)** AP2 domain of the protein encoded by the *B*. *bovis* AP2 gene BBOV_I004280, *B*. *microti* BBM_II03250, and *T*. *equi* BEWA_041620 with putative orthologues in *P*. *berghei* (PB00572.01) and *P*. *falciparum*, (PF11_0442). Both *Plasmodium* AP2 proteins are required for the development of ookinetes, and known as AP2-O. (**C)** *B*. *bovis* BBOV_II001610, *B*. *microti* BBM_II02455, and *T*. *equi* BEWA_008880 with *P*. *berghei* PB000752.01.0, a domain involved in the development of sporozoites (AP2-Sp). Predicted secondary structures for the domains of interest are depicted at the top of each of the alignments.

Recent research focused on the identification of specific AP2 proteins involved and required for regulating the expression of some stage-specific genes in *Plasmodium* [9, 10, 15, 16]. The related malaria parasites start differentiating into gametocytes while the parasites are still replicating inside erythrocytes in mammalian hosts. This crucial step requires a developmental decision, resulting in parasites that continue to replicate asexually or to differentiate into non-dividing male or female gametocytes, a life cycle event that is required to assure generation of genetic diversity and further transmission of the parasite upon mosquito acquisition. It was recently demonstrated that this developmental transition in *P*. *falciparum* parasites is regulated by the activity of the AP2 protein identified as pfAP2-g (*PFL1085w*) (Fig 4 Panel A). It was thus postulated that pfAP2-G functions as a transcriptional switch, stimulating the commitment to sexual development in this parasite [26]. Recent studies also supported the role of AP2 factors as candidate regulators driving the commitment to merozoite production in *T*. *annulata* [27]. Using a combination of techniques including transcriptome analysis and phenotypic characterization of AP2 gene knock outs, Yuda et al. [28] identified the AP2-O transcription factor, which is involved in the formation of invasive kinetes in *Plasmodium berghei* and *P*. *falciparum* (PB000572.01.0 and PF11_0442) (Fig 4, Panel B). Orthologues of the AP2-O gene have been also identified in other *Plasmodium* spp parasites. In addition, the same study also defined the sequence of the DNA involved in the binding to the AP2-O as the six-base motif TAGCTA. In a different study, Yuda et al. [29] also identified AP2-Sp (PB000752.01.0) (Fig 4, Panel C), a protein that is required for the regulation of the expression of *P*. *berghei* sporozoites and also defined the sequence TGCATG as a cis-acting element that is specific for its binding to DNA. Interestingly, the Ap2 domains involved in the binding of all these functionally defined *Plasmodium* AP2s are found to be well conserved in *B*. *bovis*, *B*. *microti*, and *T*. *equi* AP2 proteins, as shown in Fig 4. Therefore, and based on the sequence similarities of the AP2 domains shown in Fig 4, we hypothesize that the proteins encoded by genes BBOV_II005480 (~72% identity), BBM_I03085 (~76% identity), and BEWA_022490 (~77%) are functionally equivalent to the *Plasmodium* G (AP2-G) protein (PFL1085w). This is supported by previous findings demonstrating that the divergent *T*. *annulata* AP2-G protein containing AP2 motifs that are orthologous with the *P*. *falciparum* AP2-G protein are able to bind identical GxGTACxC motifs [27]. Data in S4 Table illustrates the orthologous relationships of putative AP2-G motifs of *Theileria* and *Babesia* parasites. The recently identified AP2-G *T*. *annulata* TA13515 gene [27] encodes for an AP2 motif that is 77.36% identical to the motif encoded by the functionally defined AP-G *PFL1085w* gene. However, this motif is more related in identity to the putative AP-G proteins in *Theileria parva*, *Theileria orientalis*, *T*. *equi*, *B*. *bovis*, and *B*. *microti* addressed in this study. These findings further support the testing of these AP2 as candidates for modulators in the transition of these parasites into sexual stages. Consistently, we also hypothesize that the genes identified as BBOV_I004280 (~70% identity), BBM_II03250 (~79% identity), and BEWA_041620 (~74% identity) are the functional equivalents of the *Plasmodium* AP2 proteins PF11_0442 and PB000572.01.0, which are both involved in *Plasmodium* ookinete development. Similarly, the AP2 proteins encoded by genes BBOV_II001610 (~65% identity), BBM_II02455 (~68% identity), and BEWA_008880 (~65% identity) might also be functional equivalents of AP2- Sp PB00752.01.0, which is involved in sporozoite development in malaria (Fig 4). These domain homology-driven predictions could help in prioritizing and selecting candidates for functional testing of these hypotheses, leading to define *B*. *bovis*, *B*. *microti*, and *T*. *equi* regulation pathways involved in gametocyte, ookinete, and sporozoite development.

It is possible that the proteins containing these highly conserved domains share similar DNA binding specificities among these three parasites, but this will have to be confirmed experimentally. Full transcriptome analysis in the life cycle of these organisms is not yet available, and it will be needed in order to perceive the possible role of AP2 proteins influencing life cycle transitions in these parasites.

### Conservation of genes encoding for the transcriptional regulators Myb and HMG among *B*. *bovis*, *B*. *microti*, and *T*. *equi*

The Myb proteins, which are highly conserved in eukaryotes, belong to the tryptophan cluster family and are also known to regulate gene expression. Similar to AP2 factors, Myb proteins are involved in differentiation and growth control by binding to DNA in a sequence-specific manner through a DNA-binding domain [10, 30]. Importantly, Myb proteins have been confirmed to be essential for parasite growth, cell cycle regulation, and progression in *Plasmodium* parasites [18]. *Myb* families containing eight genes each are present in the *B*. *bovis*, *T*. *equi*, and *B*. *microti* genomes (Table 3).

**Table 3:** *Myb* genes distribution among *B*. *bovis*, *B*. *microti* and *T*. *equi* genome.

Chromosome	*B*. *bovis*	*B*. *microti*	*T*. *equi*
**Chromosome 1**		BBM_I03190 BBM_I02995	BEWA_021480
**Chromosome 2**	BBOV_II005270 BBOV_II001770 BBOV_II000750	BBM_II03695	BEWA_044120 BEWA_042950
**Chromosome 3**	BBOV_III005430	BBM_III01265 BBM_III09225 BBM_III07875 BBM_III04620 BBM_III04940	BEWA_008190 BEWA_042460 BEWA_041110 BEWA_000330
**Chromosome 4**	BBOV_IV003030 BBOV_IV003940 BBOV_IV008460 BBOV_IV011350		BEWA_009170

**Fig 5:**
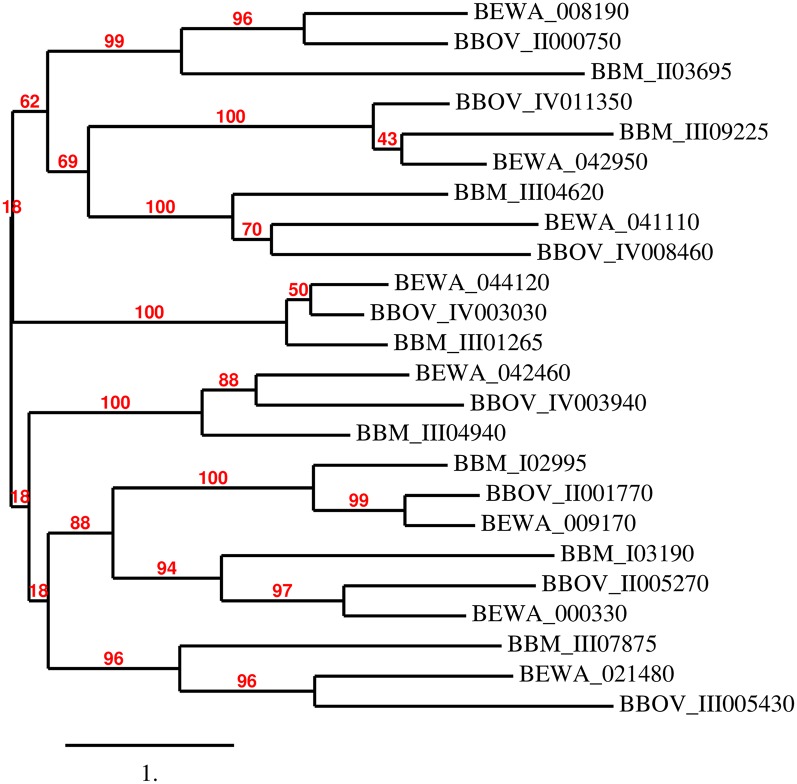
Phylogenetic relationships among the putative Myb proteins of *B*. *bovis*, *T*. *equi*, and *B*. *microti*. The unrooted phylogenetic tree was generated using the sequences of all the putative Myb proteins identified in *B*. *bovis*, *B*. *microti*, and *T*. *equi* with the program phylogeny.fr. The program calculated the branch support values in percent (%, red font) using an aLRT statistical test (http://www.phylogeny.fr/simple_phylogeny.cgi?workflow_id=ddf5cfc42f5b6c10f3df67f5152bf59a&tab_index=6&go_next=1#anchor).

Interestingly, a full set of eight *Myb* genes appears to be well conserved in sequence among the three parasites, and the Myb proteins of these three parasites appear to have similar domain architectures (S3 Fig). Their phylogenetic relationships are shown in Fig 5 and their orthologous relationships confirmed by using Bidirectional Best Blast hit analysis [31]. The orthologous Myb proteins BBOV_II001770, BEWA_009170, and BBM_I02995 contain an additional DnaJ motif located at their N-terminus region, while the DNA binding domain typical of the Myb proteins is located in their C-terminus (S3 Fig). In general, *Myb* genes are unlinked and dispersed among these three parasites’ chromosomes. However, this is not the case for the *T*. *equi Myb* genes BEWA_008190 and BEWA_008180, which are contiguous in chromosome 3 of *T*. *equi*. Protein sequence comparisons revealed limited sequence identity among the Myb proteins encoded in each of these three parasites. The possible ortholog relationships among all *Myb* genes identified in these three parasites are illustrated in the phylogenetic tree shown in Fig 5. The highly conserved gene BBOV_IV003030 encodes for a Myb protein that is 60.43% identical to the one encoded by gene BEWA_044120 in *T*. *equi*, and 50% identical to the protein encoded by gene BBM_III01265 found in in the *B*. *microti* genome. It is thus possible that these three proteins are functional homologues.

In conclusion, these relationships indicate that a core of eight *Myb* genes is conserved among these three parasites, and perhaps this is also the case in other related apicomplexan parasites as well. Consistently, searches performed on the genome of *T*. *annulata*, *T*. *parva*, and *T*. *orientalis* revealed full conservation of the set of eight *Myb* genes in these classical *Theileria* parasites (S5 Table). The complement of eight *Myb* genes from *B*. *bovis*, *B*. *microti*, and *T*. *equi* grouped in the phylogenetic tree together with the three classical *Theileria* parasites is shown in S4 Fig. It is possible to infer from these data that an ancestor organism existing previous to speciation among *Babesia* and *Theileria* also contained an eight Myb gene family.

**Fig 6:**
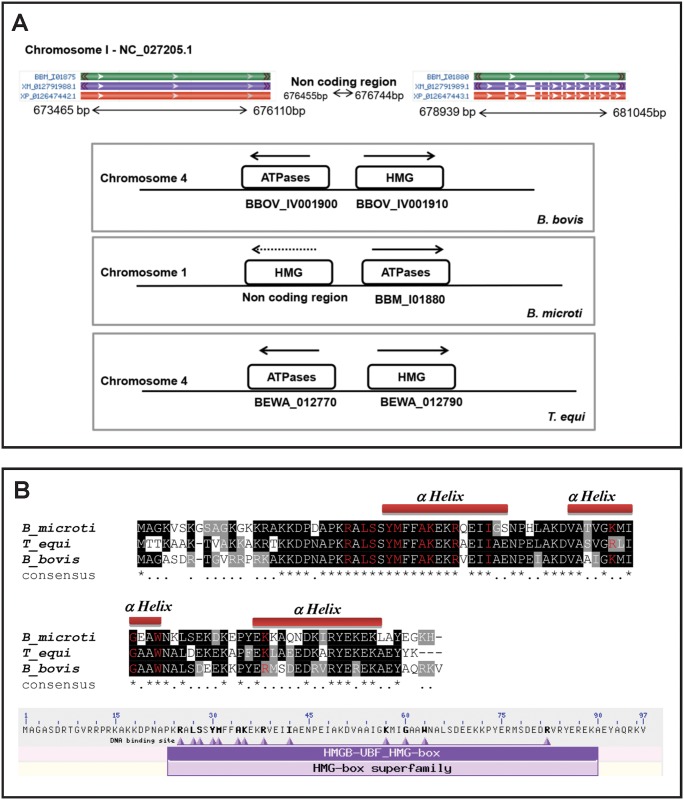
Putative HMG proteins and genes of *B*. *bovis*, *T*. *equi*, and *B*. *microti*. **(A)** *Upper*: Schematic representation of non-coding area in *B*. *microti* genome between 676455 bp and 676744 bp in chromosome 1. *Lower*: Synteny map for the *HMG* genes in the *B*. *bovis*, *B*. *microti*, and *T*. *equi*. **(B)** *Upper*: Alignment of the deduced amino acid sequences of HMG proteins identified in *B*. *bovis* (BBOV_IV001910), *T*. *equi* (BEWA_012790), and *B*. *microti* (no gene assignment). The residues in red represent the amino acids defining the HMG domain *Lower*: Location of the HMG domain and depiction of the amino acid residues defining the HMG domain (bold fonts) in the HMG protein encoded by the *B*. *bovis* gene BBOV_ IV001910.

**Fig 7:**
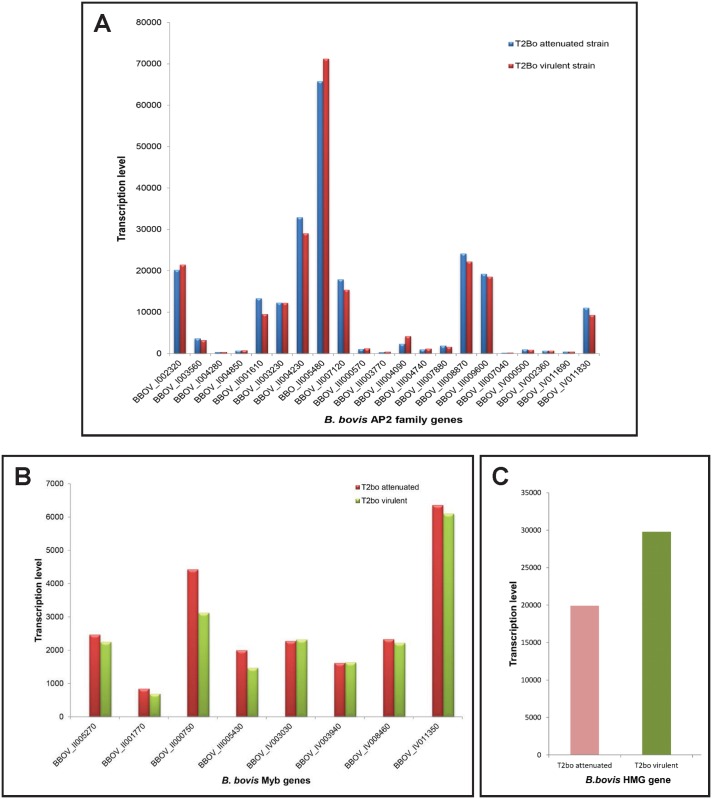
Normalized transcriptional profiles of *B*. *bovis* AP2 genes using microarray analysis in attenuated and virulent *B*. *bovis* strains. **(A)** Microarray transcriptional levels expressed as relative transcription units log cpm (copy per million) are represented in the *y*-axis, and denominations for each *B*. *bovis* AP2 gene are represented in the *x*-axis. **(B)** Transcription profile of the eight *Myb* genes identified in the virulent and attenuated T2bo strains of *B*. *bovis* using microarray analysis. **(C)** Transcription profile of the BBOV_IV001910 *HMG* gene by microarray analysis in virulent and attenuated T2Bo strain of *B*. *bovis*. The transcriptome analysis was performed in triplicate on full blood stages of parasites developing in asynchronous cultures.

The high mobility group box proteins (HMG) is a group of DNA-binding transcription factors required for the maintenance of structural alterations in DNA during transcription. The HMG superfamily is divided into three families of proteins according to their functional motifs, known as HMGA, interacting with the AT hook; HMGN, involved with nucleosomes; and HMGB, containing one or several copies of HMG box DNA binding domain [20]. In contrast to the AP2 and Myb proteins, the HMG proteins have the ability to bind A-T—rich regions of DNA rather than sequence-specific targets, in a process mediated by basic amino-acid residues of the proteins [31]. There appears to be just one *HMG* gene in *B*. *bovis* (BBOV_IV001910) in chromosome 4. This *HMG* gene has been previously cloned and characterized in yeast and *B*. *bovis* [32, 33]. The size of the predicted protein, domain and secondary structure predictions, and sequence comparisons indicate that the *B*. *bovis* BBOV_IV001910 gene is similar to the Pf *HMGB* genes [20] and, thus, it can be considered as a member of the HMGB family. The binding specificity of the *P*. *falciparum* HMGB proteins to four-way DNA junctions was also previously established [20]. In addition, a single *HMG* gene copy in *T*. *equi* BEWA_012790 was found on chromosome 4. The *B*. *bovis* BBOV_IV001910 and the *T*. *equi* BEWA_012790 predicted proteins are 65% identical and contain just 92 amino acids and a single HMG domain, lacking the typical acidic C-terminal tail [20, 33]. This putative *HMG* gene is well conserved among apicomplexans [20] and in other cells but was not annotated as such in the *B*. *microti* genome. However, BLAST analysis of the *B*. *microti* genome with the BBOV_IV001910 sequence demonstrated the occurrence of a gene present in an unannotated region of the genome (http://protists.ensembl.org/Babesia_microti_strain_ri/Tools/Blast?db=core), encoding for a homologous HMG protein. This novel putative *HMG* gene is located in the ~2829bp non-coding region between bp 676455 and 676744 of chromosome 1 of *B*. *microti* (Fig 6A upper part). Furthermore, synteny among *B*. *microti*, *B*. *bovis*, and *T*. *equi* in genomic regions encoding this gene was identified (Fig 6A bottom part). Similar to *B*. *bovis* and *T*. *equi*, the non-coding region of *B*. *microti*, which contains the HMG domain, was found to be followed by gene BBM_I01880 (Fig 6A bottom part) encoding for a protein containing an AAA domain [cd00009], an ATP binding motif present in ATPases (Fig 6A bottom part). Furthermore, we also found conservation and consistent synteny of the *HMG* gene in other related apicomplexa (*T*. *annulata*, *T*. *parva*, *P*. *falciparum*, *Plasmodium knowlesi*, and *Plasmodium vivax*) (S5). In Fig 6B, the defining amino acids for the HMG domain are shown, as well as a sequence alignment of three putative HMG proteins and the predicted secondary structures of *B*. *microti*, *B*. *bovis*, and *T*. *equi*. Interestingly, the predicted secondary structures for the in silico translated HMG proteins of *B*. *microti*, *B*. *bovis*, and *T*. *equi* shows three identical alpha-helixes comprising all amino acids involved in the HMG domain (Fig 6B), identical to what was described for their *Plasmodium* HMGB homologues [20]. It is likely that this conserved secondary structure is essential for access of the HMG proteins to its DNA binding target and for effecting protein function. In *P*. *falciparum*, the HMG proteins are present in the nucleus and induce DNA bending [20]. However, the binding targets and exact functions of the *Babesia* and *Theileria* HMG proteins remain to be defined. Considering these observations, together with the facts that gene BBOV_IV001910 is relatively highly expressed in *B*. *bovis* erythrocyte stages, as described below and shown in Fig 7C, and that key residues defining the HMG domain are also fully conserved in the *B*. *microti* putative protein (Fig 6A and 6B), we propose that the region in chromosome 1 of *B*. *microti* represented in Fig 6A represents a novel *HMG* gene. If the presence of an *HMG* gene in *B*. *microti* is confirmed experimentally, then annotation in this region of chromosome 1 of *B*. *microti* should be revised.

### Pattern of transcription of the *AP2*, *Myb*, and *HMG* genes during blood stages of *B*. *bovis*

Studies in *Plasmodium*, *T*. *annulata*, and *Toxoplasma* indicated that most AP2 genes are differentially expressed during the life cycle of the parasites [27]. *B*. *bovis* parasites have a complex life cycle involving at least two distinct hosts, the mammal bovine and arthropod tick hosts. *B*. *bovis* parasites developing in the bovine hosts only invade and reproduce in erythrocytes, and it remains unclear whether they start committing into gametogenesis while residing in the erythrocyte. However, the life stages of the parasite developing in the definitive tick vector appear to be more diverse and complex, including sexual stages and sexual reproduction, in addition to the development of kinete and sporozoite stages. Furthermore, because of their trans-ovarian mode of transmission, *Babesia* parasites are able to survive in additional stages of the tick host (adult, egg, larva, and nymph, with each of these tick stages occurring in dramatically distinct physical surroundings). We propose that this feature reflects a high degree of plasticity for this parasite, which enables radical adaptive morphological transitions during changing temperatures, surviving the non-adaptive immune system of the tick and other variable environmental factors while replicating in the tick. Based on the known role of AP2 proteins in related apicomplexans, it is possible that these changes are correlated with unique patterns of expression of AP2 proteins, in order to fulfill their role as stage-specific transcriptional regulators. Analysis of the currently available transcriptome of *B*. *bovis* in the blood stages supports this notion, as, at least, expression of two of the AP2 genes, such as BBOV_II005480 and BBOV_II004230, are significantly elevated in blood stage parasites of attenuated and virulent *B*. *bovis* T2Bo strains, while some of the AP2 genes are silenced (Fig 7A). Interestingly, and as shown in Fig 4, sequence comparisons suggest that the AP2 gene BBOV_II005480, highly transcribed in blood stages of *B*. *bovis*, is a possible correlate of the *P*. *falciparum* gene AP2-G (PFL_1085w), which was shown to be involved in the transition of *P*. *falciparum* blood stage parasites into sexual forms [26, 34]. It was recently shown that PFAP2-G functions as a master regulator controlling sexual-stage differentiation decision in *Plasmodium* parasites [26].

It is currently unknown whether the *B*. *bovis* AP2 gene BBOV_II005480 is also involved in the regulation of the expression of genes involved in sexual stage transitions and whether such stage transition also occurs in blood-stage parasites of *B*. *bovis*. However, the general currently accepted paradigm is that commitment of *B*. *bovis* to sexual forms might start with the formation of pre-gametes while the parasites reside in the bovine hosts [35, 36], which would be associated with the high level of expression of the AP2 gene BBOV_II005480 gene in the blood stages of the parasite. It is possible that *B*. *bovis* blood-stage parasites need to be primed before developing into sexual stage while still developing into the mammalian host, but this remains unknown. Alternatively, it is also possible that the expression of the gene BBOV_II005480 in blood stages is required for functions unrelated to sexual stage development. Other AP2 genes found to be highly expressed in blood stage parasites include BBOV_II004230, BBOV_III008870, BBOV_I002320, and BBOV_III009600. Interestingly, levels of transcription for the putative gene AP2-O (BBov_I004280) are negligible in the blood stage, whereas the levels of transcript for the putative AP2-Sp gene, although higher than AP2-O (BBov_II001610), are also significantly lower than AP2-G (BBOV_II005480). It could be predicted that the levels of expression of both genes are elevated in tick stages of *B*. *bovis*, as its differentiation to kinete and sporozoite stages occurs in the tick.

Comparative multistage global transcriptome analysis, together with proteomic analysis, remains to be performed in order to fully understand the patterns of expression of the *Babesia* AP2 genes among its different life stages. Taken together, these studies should provide a framework for deciphering the gene regulation networks operating during the life cycle of *B*. *bovis* and may also contribute to the design of novel methods for the control of this parasite.

*Myb* transcript analysis performed on two distinct *B*. *bovis* strains (T2bo attenuated and virulent) shows that seven out of the eight gene members are transcribed at relatively low levels in *B*. *bovis* blood stages (Fig 7B), whereas the *Myb* gene BBOV_IV011350 appears to be expressed at significantly higher levels, and, thus, members of this family are also differentially expressed by the parasite. In addition, the *HMG* gene BBOV_IV001910 is also consistently and relatively highly expressed in the two distinct *B*. *bovis* strains analyzed (T2bo attenuated and virulent strains) (Fig 7C).

**Fig 8:**
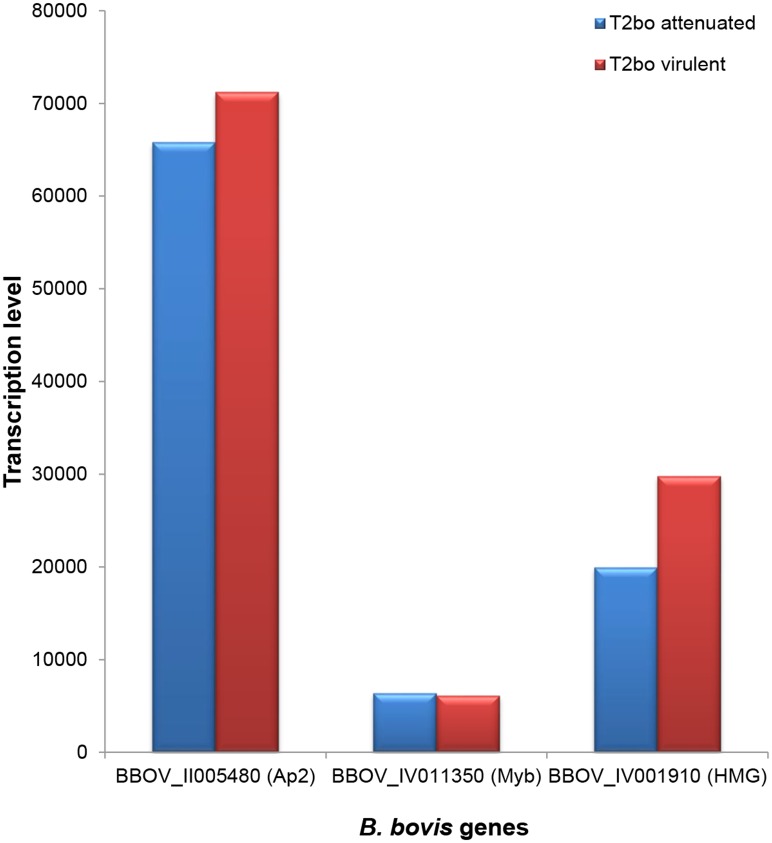
Comparison among the highest transcribed genes of the AP2, *Myb*, and *HMG* genes of *B*. *bovis*. The Ap2 gene BBOV_II005480 shows the highest transcription levels compared to the highest transcribed genes of the *Myb* and *HMG* gene groups in *B*. *bovis*.

The relative high levels of expression of the *AP2*, *Myb*, and *HMG* genes in *B*. *bovis* blood stages can be compared in Fig 8. Transcripts of the AP2 gene BBOV_II005480 were detected at levels that are at least an order of magnitude higher than the *Myb* gene and at twice the levels of the highest expressed *HMG* gene BBOV_IV001910. The functional significance of these observations remains unknown and requires further study.

However, microarray data does not show significant differences in the level of expression of the genes analyzed in this study among the attenuated and virulent strain pairs so far analyzed. There is no experimental evidence supporting the hypothesis that differential expression of these regulatory genes has any correlation with the virulence phenotype of *Babesia* strains.

## Concluding Remarks

Described here is the structure of the AP2 genes of *B*. *bovis* as well as the general organization of this family in the related *T*. *equi* and *B*. *microti* parasites. AP2 genes that are differentially expressed during the blood stages were identified and, based on domain sequence similarities, correlated with already functionally characterized *Plasmodium* AP2 proteins. A previously unknown gene family with an eight-gene core encoding for proteins, including the DNA binding domain that is characteristic for the transcription factors, known as Myb, was found conserved in *B*. *bovis*, *B*. *microti*, and *T*. *equi*. Remarkably, a conserved *HMG* gene was also described in these three parasites for the first time, although expression of the *B*. *microti HMG* gene identified in this study remains to be confirmed experimentally. The *Myb* and *HMG* genes of *B*. *bovis* might also be differentially expressed in the blood stages of the parasite. The pattern of expression of *AP2*, *Myb*, and *HMG* genes in multiple *B*. *bovis*, *T*. *equi*, and *B*. *microti* parasite stages should also be compared in order to start unraveling mechanisms involved in the regulation of gene expression in these parasites. Overall, the findings described in this study suggest conservation of regulatory genes in the face of large divergence of genome size, content and organization, and host specificities among these three apicomplexan parasites. Taking advantage of transfection and gene editing techniques, it is now possible to design KO and overexpression studies aimed at defining the resulting phenotype of mutated or genetically altered transfected parasites, leading to a correlation between gene and protein function for the AP2, HMG, and Myb proteins. In addition, experiments leading to the identification of the binding specificities for each of the *B*. *bovis*, *B*. *microti*, and *T*. *equi* AP2 proteins, as well as the Myb and HMG transcription factors, should also be performed. Finally, the ability to genetically manipulate genes encoding for transcription factors should result in a better understanding of the biology of these parasites and to the rational design of attenuated and non-tick transmissible parasite strains that can be used for the development of the next generation of live attenuated vaccines and chemotherapeutics. Conservation of key gene regulation mechanisms may lead to future development of novel converging control strategies that can be applied to apicomplexan parasites.

**Key Learning Points**

- The functional domains and general architecture of the ApiAP2, Myb, and HMG proteins remain conserved among *Babesia bovis*, *B*. *microti*, and *Theileria equi*.
- A repertoire of eight *Myb* genes is conserved among *Babesia bovis*, *B*. *microti*, and *Theileria equi*.
- Transcriptome analysis suggests that the pattern of transcription of the regulatory *AP2*, *HMG*, and *Myb* genes in *B*. *bovis* is stage specific.
- A new putative HMG gene is described for *B*. *microti*.
- Defining the functional role of regulatory genes may contribute to the development of novel convergent strategies for improved control of babesiosis and equine piroplasmosis.

**Key Papers in the Field**

1. Yuda M, Iwanaga S, Shigenobu S, Mair GR, Janse CJ, Waters AP, et al. Identification of a transcription factor in the mosquito-invasive stage of malaria parasites. Molecular microbiology. 2009;71(6):1402–14. Epub 2009/02/18. doi: 10.1111/j.1365-2958.2009.06609.x. 19220746.
2. Yuda M, Iwanaga S, Shigenobu S, Kato T, Kaneko I. Transcription factor AP2-Sp and its target genes in malarial sporozoites. Molecular microbiology. 2010;75(4):854–63. Epub 2009/12/23. doi: 10.1111/j.1365-2958.2009.07005.x. 20025671.
3. Iwanaga S, Kaneko I, Kato T, Yuda M. Identification of an AP2-family protein that is critical for malaria liver stage development. PLoS ONE. 2012;7(11):e47557. Epub 2012/11/13. doi: 10.1371/journal.pone.0047557. 23144823; PubMed Central PMCID: PMCPmc3492389.
4. Tuteja R, Ansari A, Chauhan VS. Emerging functions of transcription factors in malaria parasite. Journal of biomedicine & biotechnology. 2011;2011:461979. Epub 2011/12/02. doi: 10.1155/2011/461979. 22131806; PubMed Central PMCID: PMCPmc3216465.
5. Pieszko M, Weir W, Goodhead I, Kinnaird J, Shiels B. ApiAP2 Factors as Candidate Regulators of Stochastic Commitment to Merozoite Production in *Theileria annulata*. PLoS Negl Trop Dis. 2015;9(8):e0003933. Epub 2015/08/15. doi: 10.1371/journal.pntd.0003933. 26273826; PubMed Central PMCID: PMCPmc4537280.

## Supporting Information

S1 Fig Sequence alignment of the 26 AP2 domains identified in the 22 *B*. *microti* in silico translated AP2 proteins. Gene denominations are indicated on the left. Conserved residues are indicated in grey and black highlight.
(TIF)

S2 Fig Sequence alignment of the 24 AP2 domains identified in the 22 *T*. *equi* in silico translated AP2 proteins. Gene denominations are indicated on the left.
(TIF)

S3 Fig Myb domain distribution among the Myb proteins of the three parasites. The three parasites’ Myb proteins have similar domain architectures.
(PDF)

S4 Fig Phylogenetic tree depicting the sequence relationships among the in silico translated eight Myb proteins of *B*. *bovis*, *B*. *microti*, *T*. *equi*, *T*. *annulata*, *T*. *parva*, and *T*. *orientalis*. The orthologous genes grouped into eight groups consisting of one gene from each organism.
(TIFF)

S5 Fig Synteny map for the *HMG* genes in the *T*. *annulata*, *T*. *parva*, *P*. *Falciparum*, *P*. *knowlesi*, and *P*. *vivax*. The black arrow indicates the orientation of the genes in the chromosome.
(TIFF)

S1 Table Identity matrix generated with the amino acid sequences derived from the 22 *B*. *bovis* AP2 proteins. (DOCX)

S2 Table Features of Ap2 genes identified in the *T*. *equi* genome. (DOCX)

S3 Table Features of Ap2 genes identified in the *B*. *microti* genome. (DOCX)

S4 Table The identity percent among the putative AP2-G motifs of *Theileria* and *Babesia* parasites. (DOCX)

S5 Table *Myb* genes identified in the *T*. *annulata*, *T*. *parva*, and *T*. *orientalis* genomes. (DOCX)
