# Supplementary figures and images for: Comparative Bioinformatics Analysis of Transcription Factor Genes Indicates Conservation of Key Regulatory Domains among Babesia bovis, Babesia microti, and Theileria equi
Source: PLoS Negl Trop Dis. 2016 Nov 10;10(11):e0004983. doi: 10.1371/journal.pntd.0004983 (PMC5104403; doi:10.1371/journal.pntd.0004983)

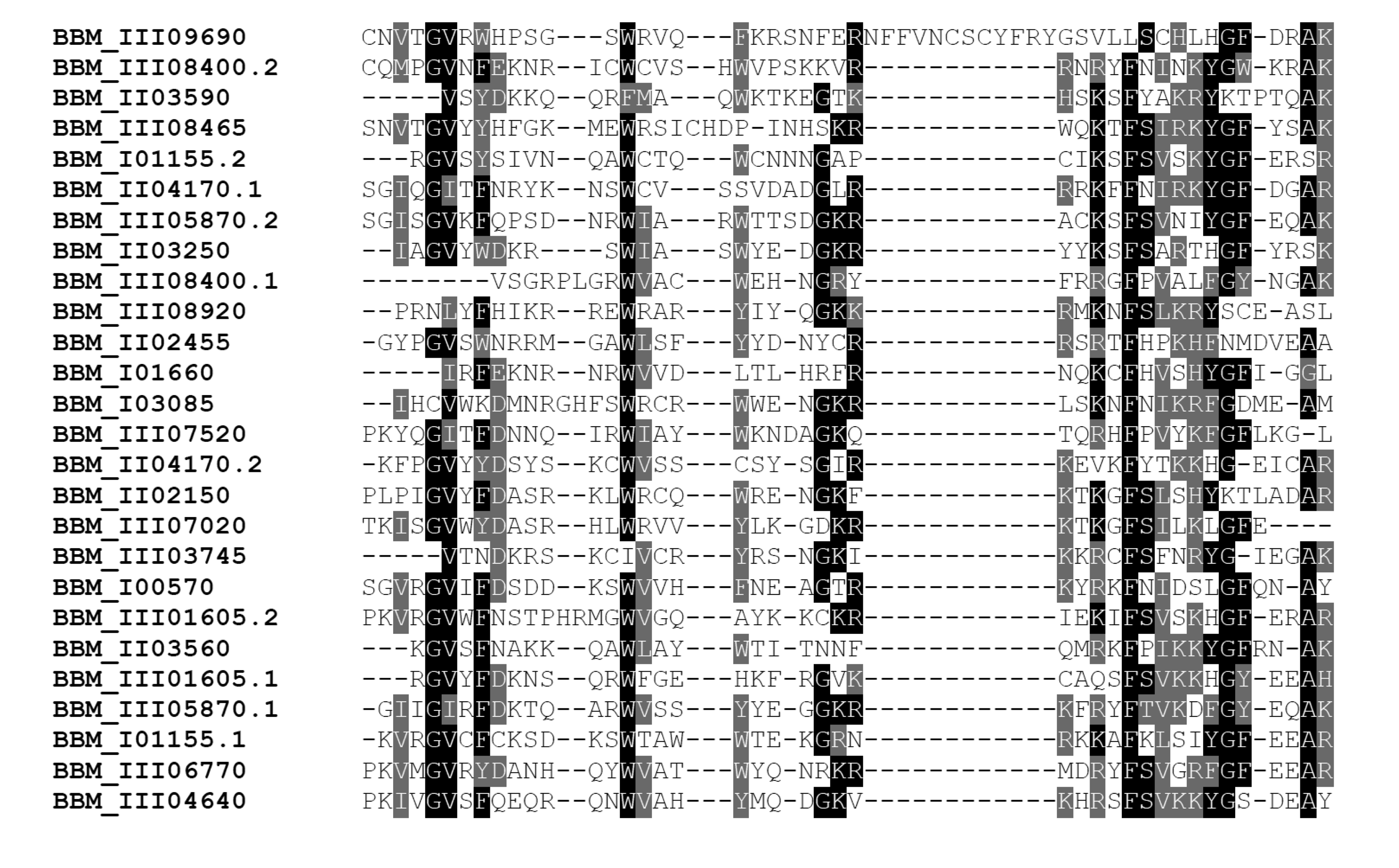

Supplement: S1 Fig — Gene denominations are indicated on the left. Conserved residues are indicated in grey and black highlight. (TIF) [file pntd.0004983.s001.tif]

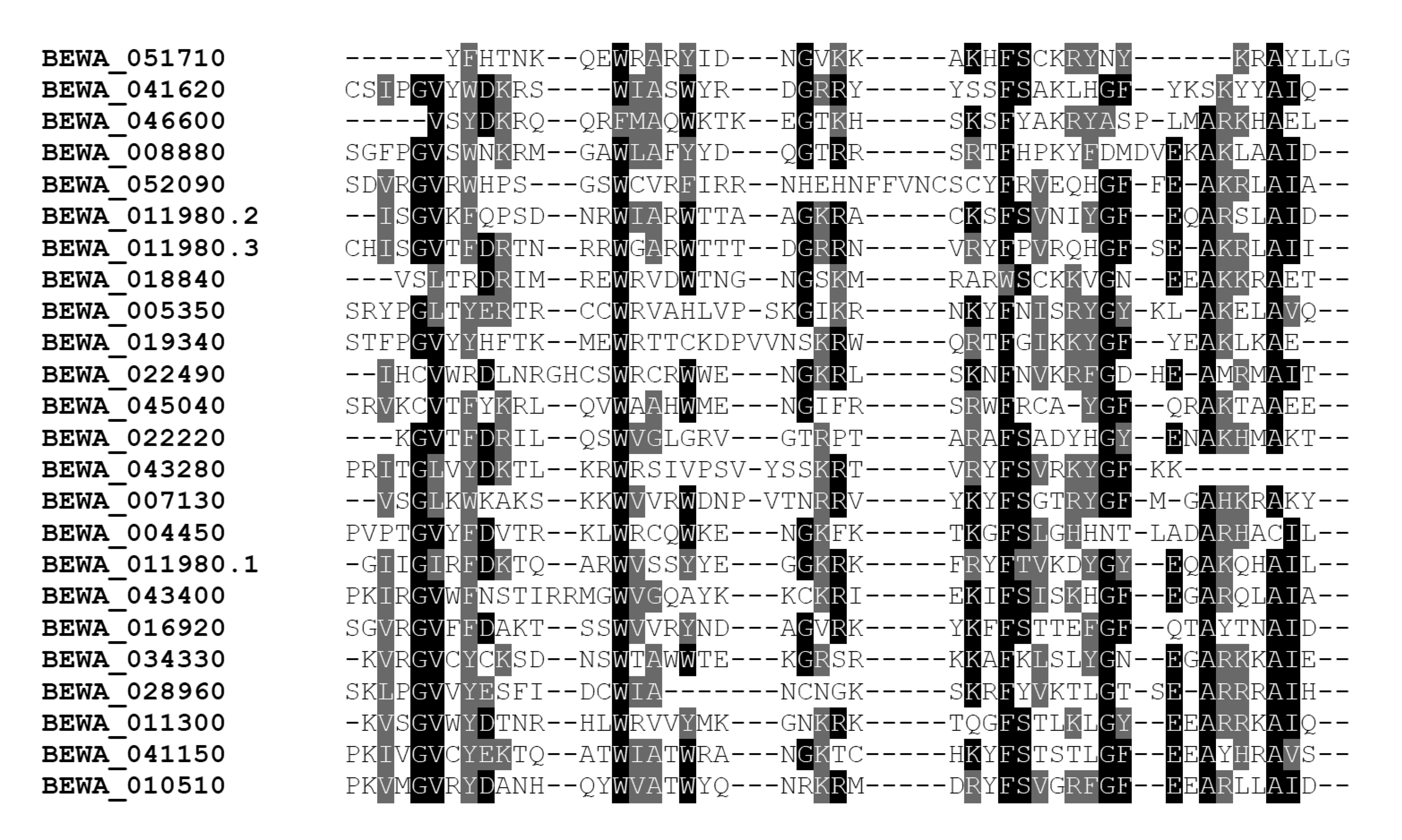

Supplement: S2 Fig — Gene denominations are indicated on the left. (TIF) [file pntd.0004983.s002.tif]

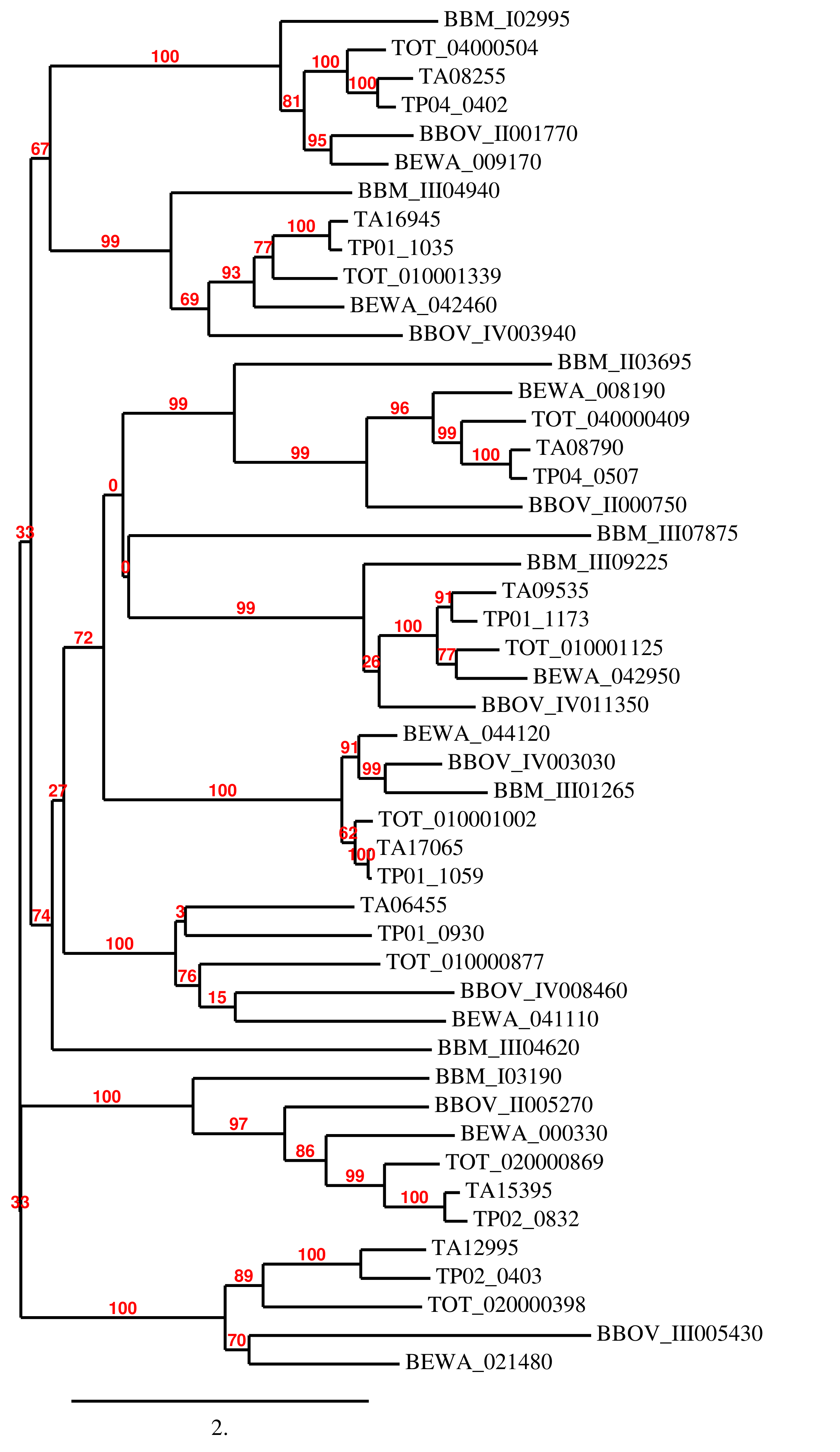

Supplement: S4 Fig — The orthologous genes grouped into eight groups consisting of one gene from each organism. (TIFF) [file pntd.0004983.s004.tiff]

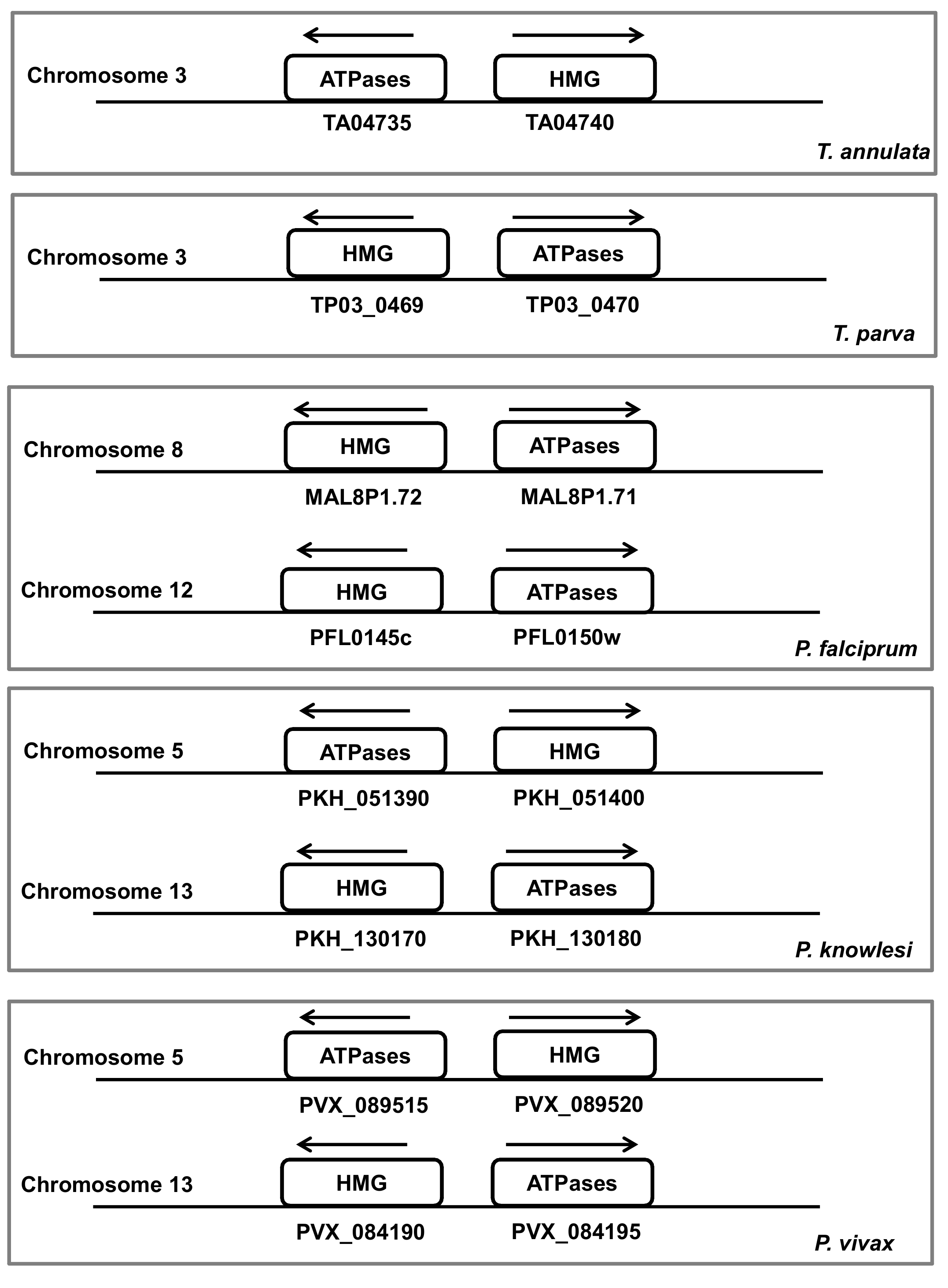

Supplement: S5 Fig — The black arrow indicates the orientation of the genes in the chromosome. (TIFF) [file pntd.0004983.s005.tiff]
