# Supplementary material for: Comparative Bioinformatics Analysis of Transcription Factor Genes Indicates Conservation of Key Regulatory Domains among Babesia bovis, Babesia microti, and Theileria equi
Source: PLoS Negl Trop Dis. 2016 Nov 10;10(11):e0004983. doi: 10.1371/journal.pntd.0004983 (PMC5104403; doi:10.1371/journal.pntd.0004983)

**>BBOV_II005270**

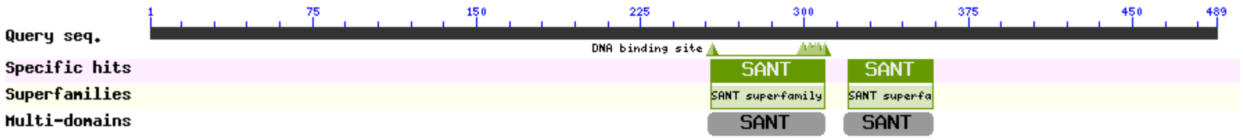


>BEWA_000330

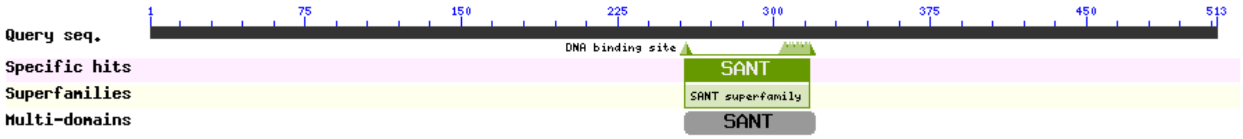


> BBM_I03190

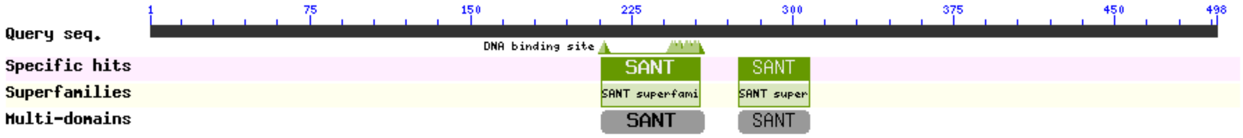

**>BBOV_II001770**

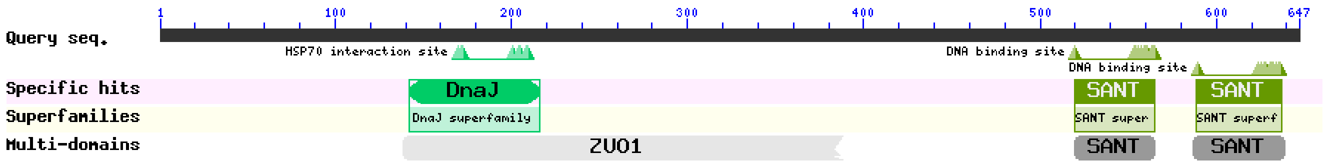


>BEWA_009170

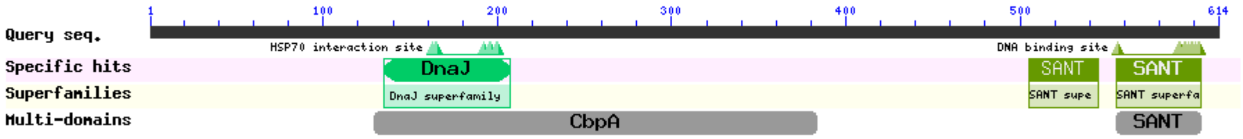


>BBM_I02995

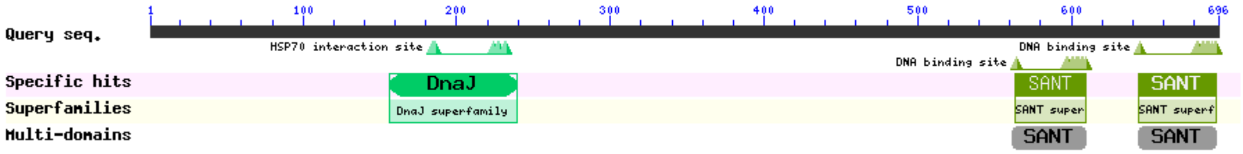

**>BBOV_II000750**

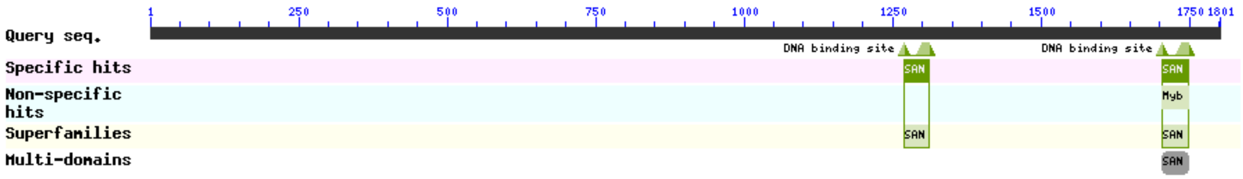


>BEWA_008190

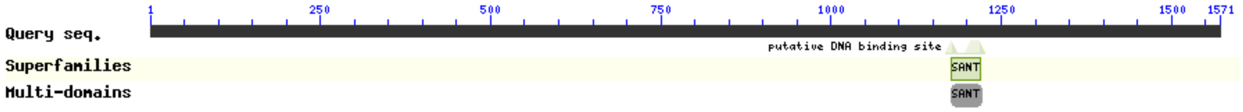


>BBM_II03695

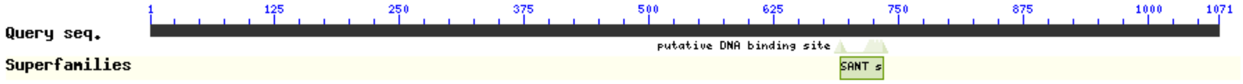

**>BBOV_III005430**

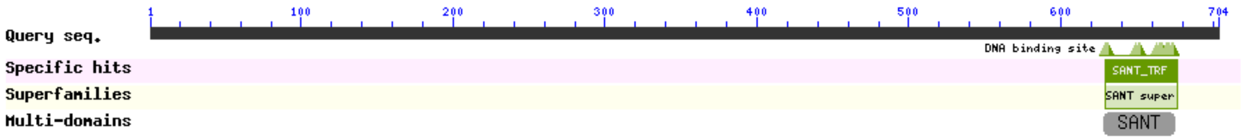


>BEWA_021480

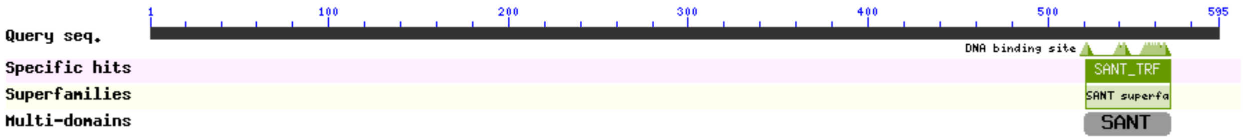


> BBM_III07875

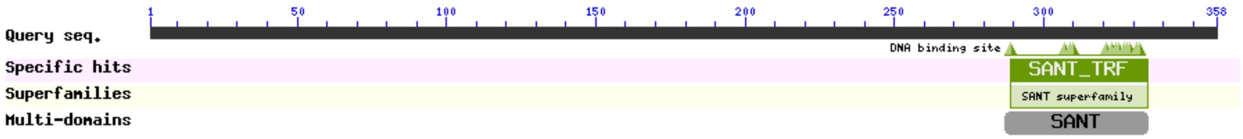

**>BBOV_IV003030**

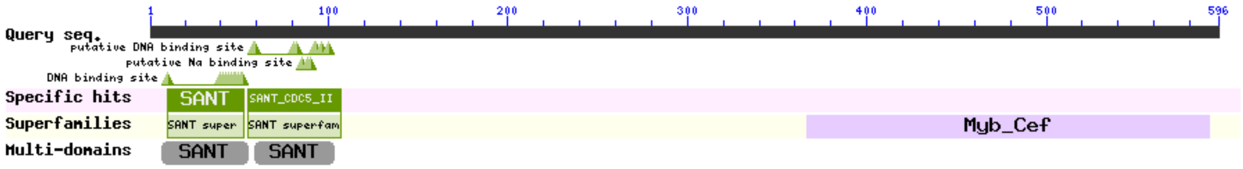


>BEWA_044120

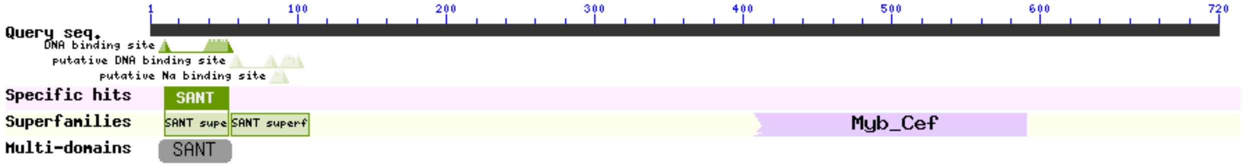


>BBM_III01265

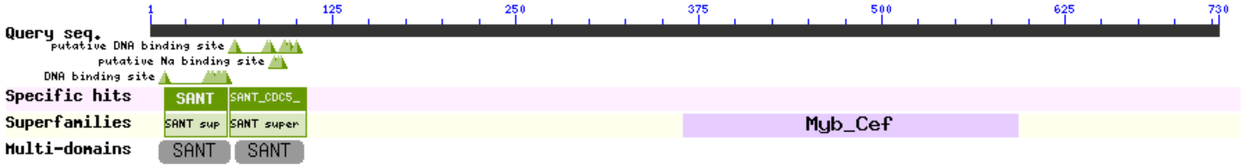

**>BBOV_IV003940**

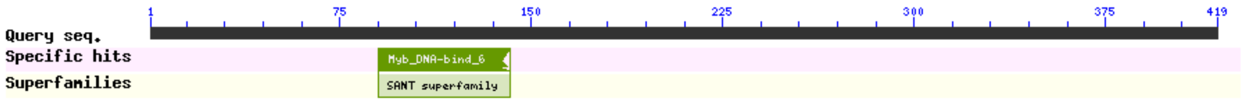


>BEWA_042460

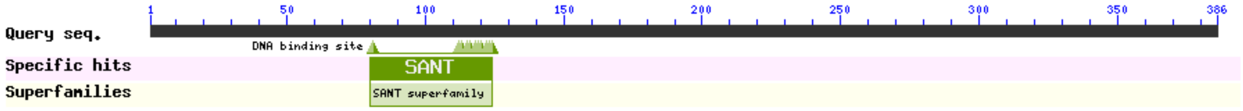


>BBM_III04940

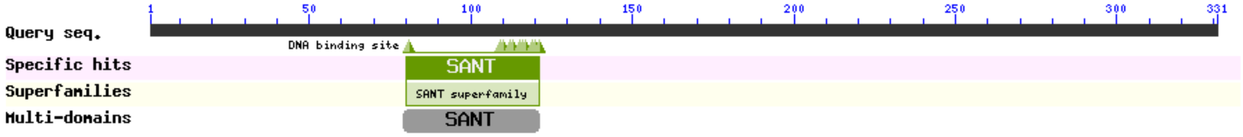

**>BBOV_IV008460**

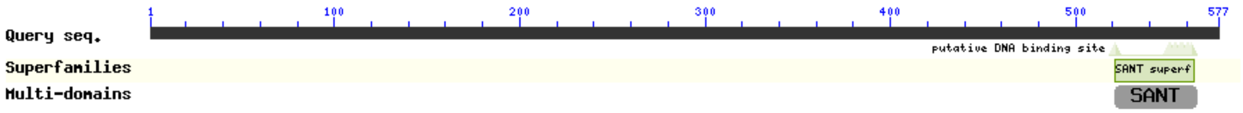


>BEWA_041110

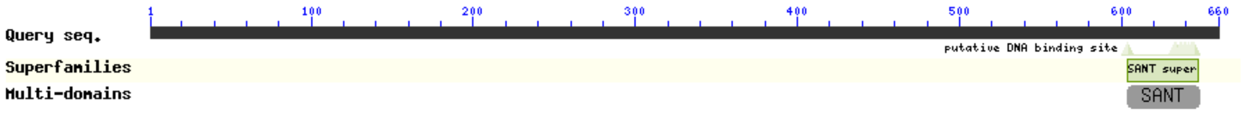


>BBM_III04620

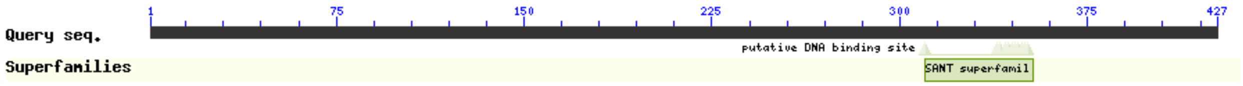

**>BBOV_IV011350**

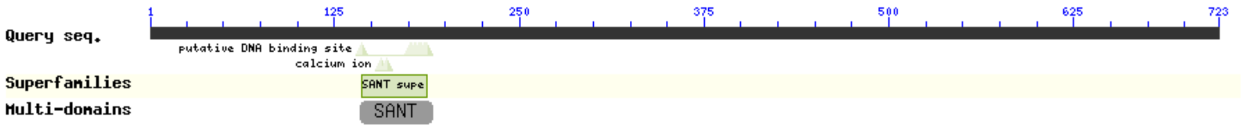


>BEWA_042950

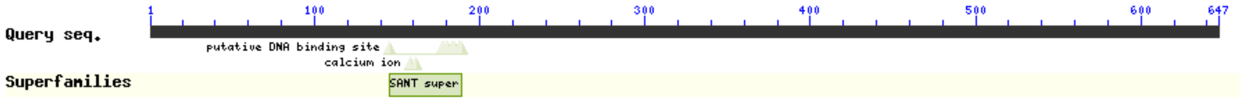


>BBM_III09225

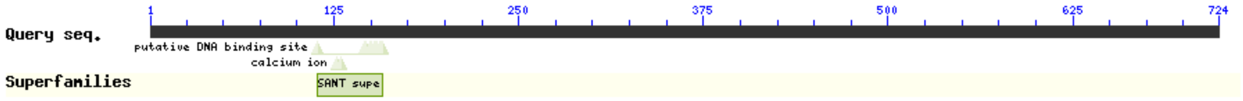

Supplement: S3 Fig — The three parasites’ Myb proteins have similar domain architectures. (PDF) [file pntd.0004983.s003.pdf]
