# Supplementary material for: Comparative Bioinformatics Analysis of Transcription Factor Genes Indicates Conservation of Key Regulatory Domains among Babesia bovis, Babesia microti, and Theileria equi
Source: PLoS Negl Trop Dis. 2016 Nov 10;10(11):e0004983. doi: 10.1371/journal.pntd.0004983 (PMC5104403; doi:10.1371/journal.pntd.0004983)
Supplement: S1 Table — (DOCX) [file pntd.0004983.s006.docx]

|  | **1** | **2** | **3** | **4** | **5** | **6** | **7** | **8** | **9** | **10** | **11** | **12** | **13** | **14** | **15** | **16** | **17** | **18** | **19** | **20** | **21** | **22** |
| --- | --- | --- | --- | --- | --- | --- | --- | --- | --- | --- | --- | --- | --- | --- | --- | --- | --- | --- | --- | --- | --- | --- |
| **1.BBOV_IV002360** | 100 | 19.72 | 16.48 | 19.4 | 14.02 | 16.39 | 12.35 | 14.57 | 9.57 | 10.71 | 10.87 | 10.28 | 13.51 | 11.89 | 12.5 | 13.98 | 10.39 | 10.26 | 6.02 | 5.83 | 6.63 | 5.88 |
| **2.BBOV_II004230** | 19.72 | 100 | 20.44 | 16.19 | 11.23 | 11.23 | 7.46 | 17.09 | 15.57 | 16.25 | 16.59 | 14.29 | 16.2 | 14.78 | 14.49 | 16.3 | 10.91 | 14.69 | 16.95 | 14.5 | 10.65 | 17.5 |
| **3.BBOV_IV000500** | 16.48 | 20.44 | 100 | 20.8 | 13.84 | 12.82 | 8.91 | 11.49 | 12.09 | 12.54 | 12.69 | 10.9 | 11.29 | 12.37 | 14.18 | 14.24 | 12.87 | 21.97 | 12.54 | 13.92 | 11.51 | 11.68 |
| **4.BBOV_III007880** | 19.4 | 16.19 | 20.8 | 100 | 21.76 | 18.57 | 16.05 | 11.6 | 10.11 | 10.23 | 12.63 | 12.81 | 6.96 | 9.16 | 16.87 | 11.92 | 8.92 | 9.4 | 11.34 | 12.44 | 13.79 | 8.06 |
| **5.BBOV_II005480** | 14.02 | 11.23 | 13.84 | 21.76 | 100 | 25.59 | 17.78 | 10.32 | 10 | 7.45 | 9.42 | 9.49 | 8.11 | 11.11 | 9.09 | 10.53 | 9.78 | 7.27 | 6.3 | 7.41 | 8.42 | 8.2 |
| **6.BBOV_I004850** | 16.39 | 11.23 | 12.82 | 18.57 | 25.59 | 100 | 21.89 | 20.97 | 0 | 7.89 | 5.41 | 7.5 | 0 | 7.53 | 0 | 6.38 | 10.71 | 0 | 5.56 | 5.13 | 9.89 | 0 |
| **7.BBOV_III004740** | 12.35 | 7.46 | 8.91 | 16.05 | 17.78 | 21.89 | 100 | 11.27 | 0 | 3.85 | 10.34 | 2.63 | 16.67 | 7.14 | 0 | 6.73 | 1.92 | 10.71 | 4.08 | 4.17 | 7.62 | 0 |
| **8.BBOV_II003230** | 14.57 | 17.09 | 11.49 | 11.6 | 10.32 | 20.97 | 11.27 | 100 | 15.62 | 15.36 | 15.04 | 10.38 | 13.46 | 18.65 | 14.18 | 12.79 | 11.05 | 18.06 | 13.56 | 12.18 | 12.64 | 14.42 |
| **9.BBOV_III007040** | 9.57 | 15.57 | 12.09 | 10.11 | 10 | 0 | 0 | 15.62 | 100 | 15.09 | 16.14 | 17.47 | 16.81 | 18.8 | 13.41 | 13.16 | 9.45 | 14.02 | 12 | 14.75 | 9.95 | 14.15 |
| **10.BBOV_I002320** | 10.71 | 16.25 | 12.54 | 10.23 | 7.45 | 7.89 | 3.85 | 15.36 | 15.09 | 100 | 19.21 | 16.1 | 13.67 | 16.62 | 19.2 | 14.72 | 11.16 | 16.03 | 13.43 | 12.88 | 12.12 | 7.96 |
| **11.BBOV_IV011690** | 10.87 | 16.59 | 12.69 | 12.63 | 9.42 | 5.41 | 10.34 | 15.04 | 16.14 | 19.21 | 100 | 19.17 | 15.38 | 21.62 | 19.89 | 15.35 | 13.33 | 16.2 | 15.46 | 8.57 | 13.51 | 14.68 |
| **12.BBOV_II007120** | 10.28 | 14.29 | 10.9 | 12.81 | 9.49 | 7.5 | 2.63 | 10.38 | 17.47 | 16.1 | 19.17 | 100 | 18.84 | 19.6 | 16.89 | 11.25 | 13.1 | 15.13 | 12.96 | 12.2 | 13.31 | 12.08 |
| **13.BBOV_I000100** | 13.51 | 16.2 | 11.29 | 6.96 | 8.11 | 0 | 16.67 | 13.46 | 16.81 | 13.67 | 15.38 | 18.84 | 100 | 21.56 | 21.28 | 15.86 | 13.54 | 16.49 | 13.76 | 20 | 16.13 | 23.68 |
| **14.BBOV_III009600** | 11.89 | 14.78 | 12.37 | 9.16 | 11.11 | 7.53 | 7.14 | 18.65 | 18.8 | 16.62 | 21.62 | 19.6 | 21.56 | 100 | 14.29 | 15.88 | 10.59 | 17.29 | 17.96 | 20 | 11.9 | 15.31 |
| **15.BBOV_II001610** | 12.5 | 14.49 | 14.18 | 16.87 | 9.09 | nan | 0 | 14.18 | 13.41 | 19.2 | 19.89 | 16.89 | 21.28 | 14.29 | 100 | 22.86 | 19.83 | 18.38 | 16.56 | 14.47 | 16.33 | 15.6 |
| **16.BBOV_I004280** | 13.98 | 16.3 | 14.24 | 11.92 | 10.53 | 6.38 | 6.73 | 12.79 | 13.16 | 14.72 | 15.35 | 11.25 | 15.86 | 15.88 | 22.86 | 100 | 17.44 | 23.57 | 21.99 | 19.73 | 19.18 | 18.18 |
| **17.BBOV_III004090** | 10.39 | 10.91 | 12.87 | 8.92 | 9.78 | 10.71 | 1.92 | 11.05 | 9.45 | 11.16 | 13.33 | 13.1 | 13.54 | 10.59 | 19.83 | 17.44 | 100 | 18.47 | 16.83 | 10.22 | 15.09 | 16.95 |
| **18.BBOV_III000570** | 10.26 | 14.69 | 21.97 | 9.4 | 7.27 | nan | 10.71 | 18.06 | 14.02 | 16.03 | 16.2 | 15.13 | 16.49 | 17.29 | 18.38 | 23.57 | 18.47 | 100 | 19.32 | 19.88 | 13.97 | 18.55 |
| **19.BBOV_IV011830** | 6.02 | 16.95 | 12.54 | 11.34 | 6.3 | 5.56 | 4.08 | 13.56 | 12 | 13.43 | 15.46 | 12.96 | 13.76 | 17.96 | 16.56 | 21.99 | 16.83 | 19.32 | 100 | 23.34 | 17.31 | 17.2 |
| **20.BBOV_III008870** | 5.83 | 14.5 | 13.92 | 12.44 | 7.41 | 5.13 | 4.17 | 12.18 | 14.75 | 12.88 | 8.57 | 12.2 | 20 | 20 | 14.47 | 19.73 | 10.22 | 19.88 | 23.34 | 100 | 20.45 | 19.5 |
| **21.BBOV_I003560** | 6.63 | 10.65 | 11.51 | 13.79 | 8.42 | 9.89 | 7.62 | 12.64 | 9.95 | 12.12 | 13.51 | 13.31 | 16.13 | 11.9 | 16.33 | 19.18 | 15.09 | 13.97 | 17.31 | 20.45 | 100 | 23.67 |
| **22.BBOV_III003770** | 5.88 | 17.5 | 11.68 | 8.06 | 8.2 | 0 | 0 | 14.42 | 14.15 | 7.96 | 14.68 | 12.08 | 23.68 | 15.31 | 15.6 | 18.18 | 16.95 | 18.55 | 17.2 | 19.5 | 23.67 | 100 |

**S1 Table. Identity matrix generated with the amino acid sequences derived from the 22 *B. bovis* AP2 proteins.**
