# Supplementary material for: Comparative Bioinformatics Analysis of Transcription Factor Genes Indicates Conservation of Key Regulatory Domains among Babesia bovis, Babesia microti, and Theileria equi
Source: PLoS Negl Trop Dis. 2016 Nov 10;10(11):e0004983. doi: 10.1371/journal.pntd.0004983 (PMC5104403; doi:10.1371/journal.pntd.0004983)
Supplement: S2 Table — (DOCX) [file pntd.0004983.s007.docx]

**S3 Table: Some characteristics of Ap2 genes identified in the *T. equi* genome**

| **No.** | **Gene**  **(locus tag)** | **Annotation** | **Length**  **(gDNA/cDNA/aa)** | **No. of exons** | **No. of AP2 domain** | **Other conserved domains** | **PI/MW[KDa]** |
| --- | --- | --- | --- | --- | --- | --- | --- |
| **Chromosome 1** | BEWA_018840 | hypothetical protein | 2331 bp/ 776 aa | 1 | One | **-** | 5.25 / 88.07 |
|  | BEWA_019340 | hypothetical protein | 829 bp/162aa | 1 | One | - | 9.71/ 18.71 |
|  | BEWA_022220 | hypothetical protein | 724 bp/648 bp /215aa | 3 | One | - | 8.94/ 24.81 |
|  | BEWA_022490 | hypothetical protein | 1687 bp/541 aa | 1 | One | - | 7.17/ 61.90 |
|  | BEWA_028960 | hypothetical protein | 2148 bp/715aa | 1 | One | - | 8.48/ 79.82 |
|  | BEWA_034330 | hypothetical protein | 2013 bp /670aa | 1 | One | - | 5.00/ 75.55 |
| **Chromosome 2** | BEWA_041150 | hypothetical protein | 898 bp /221 aa | 1 | One | ACDC | 8.48/25.83 |
|  | BEWA_043280 | hypothetical protein | 1862 bp/1827 bp /608 aa | 2 | One |  | 7.62/ 70.28 |
|  | BEWA_041620 | hypothetical protein | 870 bp /289 aa | 1 | one | - | 8.16 / 33.89 |
|  | BEWA_043400 | hypothetical protein | 1674 bp/557 aa | 1 | One | ACDC | 5.20/ 64.21 |
|  | BEWA_045040 | hypothetical protein | 848 bp /734 bp /193 aa | 4 | One |  | 10.21/ 23.26 |
| **Chromosome 3** | BEWA_004450 | hypothetical protein | 3347 bp/ /1109 aa | 1 | One | - | 5.46/123.16 |
|  | BEWA_008880 | hypothetical protein | 1561 bp /1524 bp /507 aa | 2 | One | - | 6.90/ 54.8 |
|  | BEWA_005350 | hypothetical protein | 1473 bp /490 aa | 1 | One | - | 6.26/56.49 |
|  | BEWA_007130 | hypothetical protein | 1371 bp /456 aa | 1 | One | - | 5.44/50.29 |
|  | BEWA_010510 | hypothetical protein | 1779 bp /397 aa | 2 | One | ACDC | 6.11/45.42 |
|  | BEWA_011300 | hypothetical protein | 1731 bp /576 aa |  | One | PP-binding | 5.92/65.91 |
| **Chromosome 4** | BEWA_011980 | hypothetical protein | 1383 bp /460 aa | 1 | Three | - | 6.48/51.8 |
|  | BEWA_016920 | hypothetical protein | 1071 bp /971 bp /284 aa | 3 | One |  | 10.74/33.72 |
|  | BEWA_046600 | hypothetical protein | 2209 bp /731 aa | 1 | One | - | 5.12/82.83 |
|  | BEWA_051710 | hypothetical protein | 2228 bp/1959 bp /652 aa | 5 | One |  | 6.90/ 75.64 |
|  | BEWA_052090 | hypothetical protein | 765 bp/606 bp /201 aa | 5 | one |  | 9.57/24.08 |
