# Supplementary material for: Comparative Bioinformatics Analysis of Transcription Factor Genes Indicates Conservation of Key Regulatory Domains among Babesia bovis, Babesia microti, and Theileria equi
Source: PLoS Negl Trop Dis. 2016 Nov 10;10(11):e0004983. doi: 10.1371/journal.pntd.0004983 (PMC5104403; doi:10.1371/journal.pntd.0004983)
Supplement: S3 Table — (DOCX) [file pntd.0004983.s008.docx]

**S4 Table: Some characteristics of Ap2 genes identified in the *B. microti* genome**

| **No.** | **Gene**  **(locus tag)** | **Annotation** | **Length**  **(gDNA/cDNA/aa)** | **No. of exons** | **No. of AP2 domain** | **Other conserved domains** | **PI/Mw [kDa]** |
| --- | --- | --- | --- | --- | --- | --- | --- |
| **Chromosome 1** | BBM_I00570 | hypothetical protein | 840 bp /279 aa | 1 | one | - | 10.79 /32.71 |
|  | BBM_I01155 | hypothetical protein | 2010 bp /669 aa | 1 | Three | - | 7.08 / 75.778 |
|  | BBM_I01660 | hypothetical protein | 1697 bp/ 1371 bp /456 aa | 4 | One | - | 6.47 / 53.17 |
|  | BBM_I03085 | hypothetical protein | 1605 bp/ 534 aa | 1 | one | - | 5.87 / 61.06 |
| **Chromosome 2** | BBM_II02150 | hypothetical protein | 3420 bp / 1139 aa | 1 | one | - | 6.19/125.249 |
|  | BBM_II02455 | hypothetical protein | 1083 bp / 360 aa | 1 | one | - | 8.97/3929 |
|  | BBM_II03250 | hypothetical protein | 1203 bp/ 400 aa | 1 | one | - | 7.59 / 46.1 |
|  | BBM_II03560 | hypothetical protein | 1374 bp /457 aa | 1 | One | ACDC | 6.39 / 50.37 |
|  | BBM_II03590 | hypothetical protein | 1647 bp/ 548 aa | 1 | One | - | 5.71 / 62.561 |
|  | BBM_II04170 | hypothetical protein | 1401 bp / 466 aa | 1 | Two | - | 9.14 / 53.04 |
| **Chromosome 3** | BBM_III01605 | hypothetical protein | 2028 bp /675 aa | 1 | One | ACDC | 8.49 /77.3 |
|  | BBM_III03745 | hypothetical protein | 1323 bp/ 440 aa | 1 | One | - | 9.43 / 50.45 |
|  | BBM_III04640 | hypothetical protein | 3155 bp/ 2832 bp/ 943 aa | 3 | One | HATPase  HSP90 | 5.86/ 107.99 |
|  | BBM_III05870 | hypothetical protein | 2352 bp/ 1728 bp / 575 aa | 2 | Two | - | 8.66 /66.14 |
|  | BBM_III06770 | hypothetical protein | 1314 bp /437 aa | 1 | One | ACDC | 6.20 /49.57 |
|  | BBM_III07020 | hypothetical protein | 1308 bp/ 435 aa | 1 | one | - | 8.59 /49.42 |
|  | BBM_III07520 | hypothetical protein | 332 aa | 1 | One | - | 8.78/ 39.02 |
|  | BBM_III08400 | hypothetical protein | 1962 bp / 653 aa | 1 | Two | - | 9.19/ 73.54 |
|  | BBM_III08465 | hypothetical protein | 639 bp / 212 aa | 1 | one | - | 9.29 /24.83 |
|  | BBM_III08920 | hypothetical protein | 2804 bp /1905 bp/ 634 aa | 4 | One | - | 9.09/71.38 |
|  | BBM_III09690 | hypothetical protein | 1526 bp/ 1188 bp/ 395 aa | 5 | One | - | 8.82 /45.31 |
