# Supplementary material for: Comparative Bioinformatics Analysis of Transcription Factor Genes Indicates Conservation of Key Regulatory Domains among Babesia bovis, Babesia microti, and Theileria equi
Source: PLoS Negl Trop Dis. 2016 Nov 10;10(11):e0004983. doi: 10.1371/journal.pntd.0004983 (PMC5104403; doi:10.1371/journal.pntd.0004983)
Supplement: S4 Table — (DOCX) [file pntd.0004983.s009.docx]

|  |  | 1 | 2 | 3 | 4 | 5 | 6 | 7 |
| --- | --- | --- | --- | --- | --- | --- | --- | --- |
| 1 | PFL1085w[AP2-G] | 100 | 75.93 | 72.22 | 77.36 | 77.36 | 77.36 | 77.36 |
| 2 | BBM_I03085 |  | 100 | 81.48 | 84.91 | 83.02 | 83.02 | 84.91 |
| 3 | BBOV_II005480 |  |  | 100 | 94.34 | 92.45 | 92.45 | 90.57 |
| 4 | BEWA_022490 |  |  |  | 100 | 94.34 | 94.34 | 96.23 |
| 5 | TA13515 |  |  |  |  | 100 | 100 | 98.11 |
| 6 | TP02_0497 |  |  |  |  |  | 100 | 98.11 |
| 7 | TOT_020000484 |  |  |  |  |  |  | 100 |
