# Supplementary material for: Comparative Bioinformatics Analysis of Transcription Factor Genes Indicates Conservation of Key Regulatory Domains among Babesia bovis, Babesia microti, and Theileria equi
Source: PLoS Negl Trop Dis. 2016 Nov 10;10(11):e0004983. doi: 10.1371/journal.pntd.0004983 (PMC5104403; doi:10.1371/journal.pntd.0004983)
Supplement: S5 Table — (DOCX) [file pntd.0004983.s010.docx]

**S5 Table*: Myb* genes identified in the *T. annulat, T. parva* and *T. orientalis* genome.**

| Chromosome | *T.annulata* | *T. parva* | *T. orientalis* |
| --- | --- | --- | --- |
| Chromosome one | TA09535  TA06455  TA16945  TA17065 | [TP01_](http://www.ncbi.nlm.nih.gov/gene/3501486)1173  TP01_0930  TP01_1035  TP01_1059 | TOT_010001002  TOT_010001339  TOT_01000877  TOT_01001125 |
| Chromosome two | TA15395  TA12995 | [TP02_0832](http://www.ncbi.nlm.nih.gov/gene/3501486)  TP02_0403 | TOT_020000398  TOT_020000869 |
| Chromosome three |  |  |  |
| Chromosome four | TA08790  TA08255 | TP04_0507  TP04_0402 | TOT_040000409  TOT_04000504 |
